# Constrained Transmit Beampattern Design Using a Correlated LFM-PC Waveform Set in MIMO Radar

**DOI:** 10.3390/s20030773

**Published:** 2020-01-31

**Authors:** Sheng Hong, Yantao Dong, Rui Xie, Yu Ai, Yuhao Wang

**Affiliations:** 1 School of Information Engineering, Nanchang University, Nanchang 330031, China; 406130717274@email.ncu.edu.cn (Y.D.); 416114416053@email.ncu.edu.cn (Y.A.); wangyuhao@ncu.edu.cn (Y.W.); 2 School of Electronic Information and Communications, Huazhong University of Science and Technology, Wuhan 430074, China; xierui@hust.edu.cn

**Keywords:** MIMO radar, beampattern, ambiguity function, sidelobes, bi-objective optimization

## Abstract

This paper considers the design of a desired transmit beampattern under the good ambiguity function constraint using a correlated linear frequency modulation-phase coded (LFM-PC) waveform set in multiple-input-multiple-output (MIMO) radar. Different from most existing beampattern design approaches, we propose using the LFM-PC waveform set to conquer the challenging problem of synthesizing waveforms with constant-envelope and easy-generation properties, and, meanwhile, solve the hard constraint of a good ambiguity behaviour. First, the ambiguity function of the LFM-PC waveform set is derived, and the superiority of LFM-PC waveforms over LFM and PC waveforms is verified. The temporal and spatial characteristic analysis of the LFM-PC waveform set demonstrates that both the transmit beampattern and sidelobe level are mainly affected by the frequency intervals, bandwidths, and phase-coded sequences of the LFM-PC waveform set. Finally, the constrained beampattern design problem is formulated by optimizing these parameters for desired beampatterns and low sidelobes at different doppler frequencies, which is a bi-objective optimization problem. To solve this, we propose a joint optimization strategy followed by a mandatory optimization, where the sequence quadratic programming (SQP) algorithm and adaptive clonal selection (ACS) algorithm are exploited iteratively. The simulation results demonstrate the efficiency of our proposed method.

## 1. Introduction

A multiple-input-multiple-output (MIMO) radar has multiple transmit antennas to transmit multiple probing waveforms. The multiple waveforms can be orthogonal or correlated [1]. The waveform diversity offered by MIMO radar generates improved capabilities over the traditional phased-array radar in terms of target detection, identification, classification, and localization [2]. MIMO radars can be grouped into widely-distributed [3] and colocated [4] MIMO radars. We focus on the colocated MIMO radars in this paper.

Based on the ability to probe with distinct waveforms, transmit beampattern design in colocated MIMO radar has become a popular research topic [5]. By shaping the transmit beampattern, the radar radiation power can be properly managed to improve the energy efficiency, reduce the interference and increase the detection probability [6,7]. Thus, the waveforms should be optimally designed for the desired transmit beampattern, to control the radiation power distribution.

The related waveform design approaches can be divided into two categories. One category is a two-step strategy [5,8,9], where the waveform covariance matrix is first optimized, then the transmitted waveforms, under practical constraints, are synthesized using the covariance matrix. For instance, the waveform covariance matrix was devised to match the desired pattern through the semi-definite quadratic programming (SDQP) technique [5] and semi-definite programming (SDP) technique [8], and then a cyclic algorithm (CA) was proposed in [10], to synthesize the constant modulus waveform matrix to approximate the covariance matrix. Another category is to design the transmitted waveforms directly to fulfill a desired beampattern without the synthesis stage of the waveform covariance matrix [11,12,13,14,15,16]. In addition, independent waveforms [17,18] have been pre-processed with complex weights to form multi-rank beamformers to achieve the desired beampattern. However, this kind of method cannot guarantee equal power transmission from each antenna and a variety of desired beampatterns cannot be obtained.

Furthermore, the transmit beampattern design problem in more strict and practical situations is researched. Firstly, some works have paid attention to better approximating the desired beampattern by considering different aspects of the performance, such as the ripples within the energy focusing section, the attenuation of the sidelobes, the width of the transition band, the angle step-size, and the required number of transmit antennas [19]. The peak sidelobe level or the integrated sidelobe level of transmit beampattern were taken as figures of merit in the beampattern design problem [20]. Secondly, the robust design of waveform covariance matrices over steering vector mismatches and manifold vector perturbations [9,21] was studied. Thirdly, transmit beampatterns under spectral [22] and spatial interference [23] constraint have been considered. The work in [24] focused on the signal-to-interference-plus-noise ratio (SINR) enhancement using transmitted waveform covariance matrix optimization in colocated MIMO radars. The sparse frequency waveform design problem for the desired transmit beampattern in spectrum-crowded environment was considered in [25,26].

The above existing beampattern matching design methods rarely consider the following two problems. Firstly, the waveforms cannot be generated easily, as the waveforms synthesized from the designed covariance matrix must be constrained to a constant envelope. Secondly, the beampattern designs, above mentioned, might result in waveforms with high peak sidelobe levels, and, generally, with an undesired ambiguity function behaviour. Aiming at the first problem, a correlated multicarrier linear-frequency-modulation (LFM) waveform set was designed in [27], as the transmitted signals for the beampattern directly.

The obtained LFM waveforms have some advantages over the present waveforms, such as a constant-envelope and easy generation. Aiming at the second problem, the MIMO radar waveforms were synthesized for a desired beampattern in [28], under the constant modulus and similarity constraints, where the similarity constraint exploited an LFM waveform as a benchmark, thus allowing the designed signal to share good ambiguity characteristics with the known LFM waveform. However, the method in [27] did not consider the range sidelobe level or the ambiguity function of the designed waveforms, while the method in [28] still suffered from the constraint of constant-envelope and easy-generation. Therefore, we develop a new solution for the two problems in the transmit beampattern design.

It is well known that the LFM signal, which is used in [27,28], has some outstanding characteristics, such as constant-envelope, easy generation, and good doppler tolerance [29,30,31]. However, the correlated multicarrier LFM waveform set, proposed in [27], has high grating sidelobes, which was demonstrated in [32,33]. The transmit beampattern design problem, formulated in [27], did not consider the problem of sidelobes. Moreover, we found that the correlated multicarrier linear frequency modulation-phase coded (LFM-PC) waveforms are more general than the correlated multicarrier LFM waveforms, and can suppress the grating sidelobes directly [34]. Therefore, we developed the transmit beampattern design problem with the constraint of the range sidelobe levels at different doppler frequencies by using the correlated LFM-PC waveforms. Compared with the method in [28], our proposed method has the advantages of constant-envelope and easy-generation, and the proposed LFM-PC waveforms also provide a good waveform benchmark for the method in [28].

In this paper, the constrained beampattern design problem is considered by using the correlated LFM-PC waveform set. The ambiguity function of the LFM-PC waveform set is devised, and it shows that the LFM-PC waveform set can inherit the advantages of both the LFM and PC waveforms, and weaken their disadvantages. Then, being founded from the temporal and spatial characteristics of LFM-PC waveforms, the range sidelobes and transmit beampattern are both affected by the frequency intervals, bandwidths, and the phase-coded sequences. Thus, by optimally designing these three waveform parameters of the LFM-PC waveform set, the correlation properties of waveforms are controlled and adjusted, to match the desired beampattern with the constraints of range sidelobe levels at different doppler frequencies.

The optimization process includes several steps. First, according to the desired transmit beampattern, the desired waveform covariance matrix is optimized via the convex (CVX) toolbox. Then, based on the desired covariance matrix, a bi-objective optimization problem is formulated, where the first objective function is the covariance matrix matching error, and the second objective function is the maximum range peak-to-sidelobe level (PSL) in the doppler and angle space. To solve this, we propose a strategy of two optimization stages. In the first stage, the beampattern and the PSL are jointly optimized. This stage ends when the beampattern performance and PSL performance both cannot become better. In the second stage, the beampattern is mandatorily optimized with a relaxed PSL performance.

Based on the sequence quadratic programming (SQP) [35] and adaptive clonal selection (ACS) [36] algorithms, we introduce the sequential iterative algorithm to synthesize the desired beampattern by enforcing the constraint of a good ambiguity function. The iterations split the bi-objective optimization problem into two single-objective optimization problems. Finally, we evaluate the performance of the proposed algorithm via numerical simulations in terms of the iteration process of optimization, synthesized transmit beampattern, range sidelobes, and the ambiguity function of designed waveforms. Our results highlight the superiority of the proposed algorithm.

To deal with the above issues, our contributions are given below:

- The ambiguity function and characteristics of the LFM-PC waveform set are derived and analyzed, and its superiorities over both the LFM and PC waveform sets are demonstrated.
- For other beampattern design methods, the waveforms are constrained to be constant-envelope and have to be synthesized by complex algorithms with a heavy computation load. However, in our proposed method, the designed LFM-PC waveforms corresponding to the desired beampattern is naturally advantageous with a constant-envelope and easy generation.
- For other beampattern design methods, the temporal properties of the designed waveforms are rarely considered. In our proposed method, by using the LFM-PC waveform set to design the beampattern, the designed waveforms share good temporal characteristics of LFM-PC waveforms, such as a thumbtack ambiguity and low range sidelobes.
- The constrained beampattern design problem is formulated as a bi-objective optimization problem. To solve it, a novel strategy of first joint optimization then mandatory optimization is proposed. The beampattern performance is mandatorily guaranteed while the ambiguity function behaviour is also good.

The rest of this paper is organized as follows. The signal model and signal processing structure for the LFM-PC waveform set are given in Section 2. The ambiguity function of the multicarrier LFM-PC waveform set is derived and discussed in Section 3. Section 4 presents the temporal-spatial characteristics analysis. The optimization process for the constrained beampattern design problem is demonstrated in Section 5. Simulation results are given in Section 6. Finally, Section 7 concludes the paper.

## 2. The MIMO Radar Signal Model With the LFM-PC Waveform Set

### 2.1. A Set of Correlated LFM-PC Waveforms

A set of correlated LFM-PC waveforms are considered in MIMO radar, where each transmitted waveform is constructed by modulating a phase-coded (PC) sequence on the LFM waveform. Different transmitted waveforms occupy different spectrums, thus the received signal can obtain a wideband effect and high resolution through matched filter and receive beamforming.

**Figure 1:**
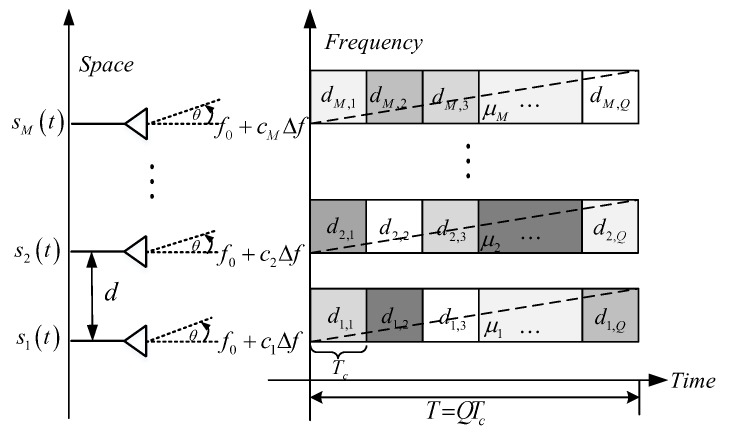
The structure of the linear frequency modulation-phase coded (LFM-PC) waveform set.

We assume that the transmitter and receiver are colocated in space, and the transmit array is a uniform linear array (ULA) consisting of *M* transmit antennas. A set of *M* transmitted waveforms are denoted by $s\left(t\right)={\left[s_{1}\left(t\right),s_{2}\left(t\right),\cdots ,s_{M}\left(t\right)\right]}^{T}$, and the waveform transmitted by the *m*th antenna can be written as

(1a) $$\begin{array}{cc} s_{m}\left(t\right) & =u_{m}^{PC}\left(t\right)e^{j\pi \mu_{m}t^{2}}\;e^{j2\pi (f_{0}+c_{m}\Delta f)t}, \end{array}$$

(1b) $$\begin{array}{cc} u_{m}^{PC}\left(t\right) & =\frac{1}{\sqrt{Q}}\sum_{q=0}^{Q-1}d_{m,q+1}rect\left(t-qT_{c}\right), \end{array}$$

(1c) $$\begin{array}{cc} rect(t) & =\left\{\begin{array}{ll} 1/\sqrt{T_{c}}, & \mathrm{if}\;0\leq t\leq T_{c}\\ 0, & \mathrm{otherwise} \end{array}\right., \end{array}$$

where 0≤t≤T and m=1,2,⋯,M. *Q* is the code length and $T_{c}$ is the code width, then the pulse duration is $T=QT_{c}$. $d_{m,q}=\exp\left(j\phi_{m,q}\right)$ denotes the *q*th code element modulated on the *m*th transmitted waveform, and $\phi_{m,q}$ is the code phase. rect(t) represents the amplitude function of the sub-pulse. $c_{m}$ is the frequency code. $\Delta f=n_{0}/T$ is the assumed frequency step, where $n_{0}$ is an arbitrary positive integer. $f_{0}$ is the carrier frequency, which is usually mixed by the center frequency, thus is usually set to be $f_{0}=0\;\mathrm{Hz}$ for the sake of simplicity. $\mu_{m}=B_{\mathrm{LFM},m}/T$ indicates the chirp rate of the *m*th LFM, and $B_{\mathrm{LFM},m}$ is the bandwidth of the *m*th LFM. As the PC is modulated on the LFM, the bandwidth of the *m*th transmitted waveform on the carrier can be calculated by $B_{m}=\sqrt{B_{\mathrm{LFM},m}^{2}+{\left(1/T_{c}\right)}^{2}}$ [37]. By denoting the frequency interval between the (m+1)th carrier frequency and the *m*th carrier frequency by $\Delta f_{m}=c_{m+1}\Delta f-c_{m}\Delta f$, the total bandwidth of the transmitted signal is $B=\sum_{m=1}^{M-1}\Delta f_{m}+B_{M}$. The structure of the LFM-PC waveform set is shown in Figure 1.

The signal model in (1) for the LFM-PC waveform set provides a general framework for both the LFM waveform set and the PC waveform set. When $d_{m,q}=1$ for ∀m,q is satisfied, Equation (1) becomes the LFM waveform set proposed in [27]. When $\mu_{m}=\mu =0$ for ∀m is satisfied, Equation (1) becomes the PC waveform set.

The correlation properties of the LFM-PC waveform set can be controlled by two kinds of waveform parameters. The first kind of waveform parameters comprises the frequency intervals (or carrier frequencies) and bandwidths. For example, if $c_{m}=m-\left(M+1\right)/2$ and the bandwidths are uniformly equal (i.e., $B_{m}=B_{s}$ or $\mu_{m}=\mu$, ∀m), Equation (1) becomes the strictly orthogonal frequency division LFM-PC (OFD LFM-PC) waveform set, and the LFM-PC waveforms in the set are mutually orthogonal. Then, it is not difficult to deduce the signal models for the OFD LFM or the OFD PC waveform set. The second kind of waveform parameters contains the phase-coded sequences, which can be presented as a column vector of phase codes $\phi ={\left[\phi_{1,1},\phi_{1,2},\cdots ,\phi_{1,Q},\cdots ,\phi_{M,1},\cdots ,\phi_{M,Q}\right]}^{T}$.

Thus, the frequency intervals $\Delta f_{m}$ (which are determined by the carrier frequencies), the bandwidths $B_{m}$, and the phase codes ϕ of the multicarrier LFM-PC waveform set can be jointly optimized to adjust the correlation properties of the waveform set. Clearly, the frequency interval $\Delta f_{m}$ is closely related with the frequency code $c_{m}$ and the frequency step Δf. The bandwidth $B_{m}$ depends on the chirp rate $\mu_{m}$ by fixing the code width $T_{c}$ here in this paper.

### 2.2. The Signal Processing Structure for LFM-PC Waveforms

By ignoring the signal attenuation during propagation, the waveform illuminated into spatial direction θ can be written as

(2) $$\begin{array}{c} s\left(\theta ,t\right)=a_{t}^{T}\left(\theta \right)s\left(t\right)=\sum_{m=1}^{M}e^{j2\pi c_{m}d\sin\theta /\lambda }s_{m}\left(t\right), \end{array}$$

where $a_{t}^{}\left(\theta \right)={\left[e^{j\pi c_{1}\sin\theta },e^{j\pi c_{2}\sin\theta },\cdots ,e^{j\pi c_{M}\sin\theta }\right]}^{T}$ denotes the transmit steering vector with the center of the array taken as the reference point, and $c_{m}=m-\left(M+1\right)/2$. Equation (2) means that coherent MIMO radar would illuminate different waveforms into different spatial directions, thus s(θ,t) is termed as an angular waveform, subsequently.

**Figure 2:**
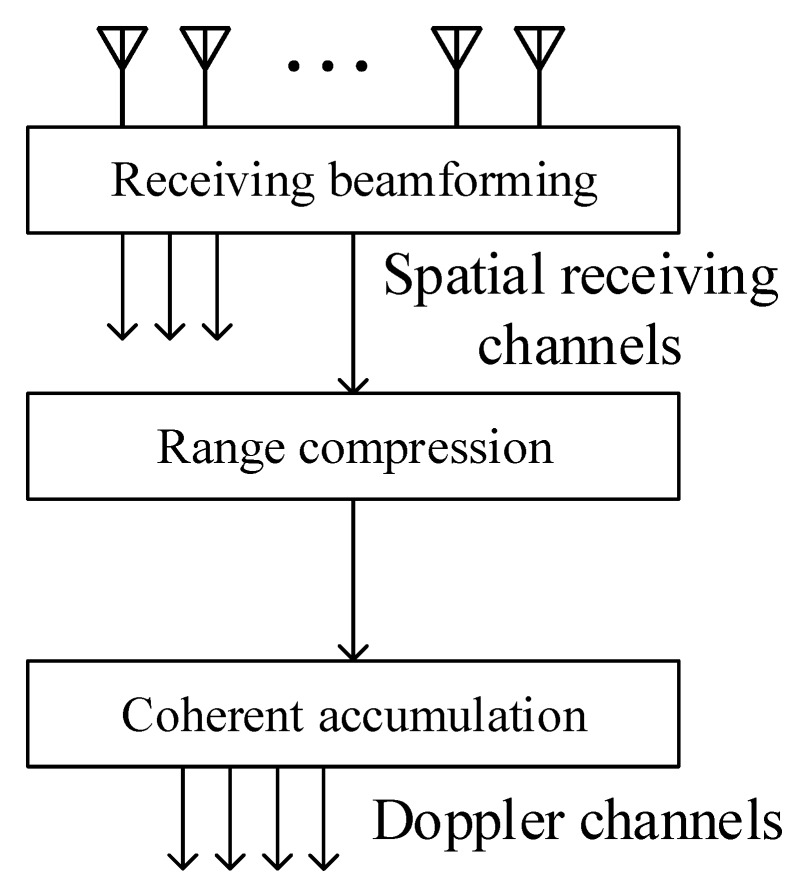
The hierarchical signal processing structure for the colocated MIMO radar.

The hierarchical signal processing structure of a colocated MIMO radar is shown in Figure 2. According to this structure, signals from receiving antennas would be first transformed into multiple spatial receiving channels, each channel characterized by an angle $\theta^{{}^{\prime }}$, where $\theta^{{}^{\prime }}$ denotes the spatial direction the spatial beam points to. Then, each spatial receiving channel is followed by a range compressor. Finally, the doppler processing is implemented by coherent accumulation.

Usually, target returns that can pass the spatial receiving channel characterized by $\theta^{{}^{\prime }}$ without amplitude attenuation would all bear the same waveform signature $a_{t}^{T}\left(\theta^{{}^{\prime }}\right)s\left(t\right)$. Thus, based on the above signal processing scheme, the ambiguity function of the angular waveform in the spatial receiving channel characterized by $\theta =\theta^{{}^{\prime }}$ can be represented as

(3) $$\begin{array}{cc} \chi (\tau ,f_{d},\theta ) & =\int_{-\infty }^{\infty }s\left(\theta ,t\right)s^{H}\left(\theta ,t-\tau \right)e^{j2\pi f_{d}t}dt\\ \; & =\;\;\;\int_{-\infty }^{\infty }\;\;\;a_{t}^{T}\left(\theta \right)s\left(t\right)s^{H}\left(t-\tau \right)a_{t}^{*}\left(\theta \right)e^{j2\pi f_{d}t}dt. \end{array}$$

The three-dimensional ambiguity function $\chi (\tau ,f_{d},\theta )$ can demonstrate the overall performance of the LFM-PC waveform set under the signal processing structure in Figure 2. $\chi (\tau ,f_{d},\theta )$ can present the sidelobe properties in the time delay τ-dimension and the doppler frequency $f_{d}$-dimension, which can be regarded as the temporal ambiguity function of the transmitted angular waveforms at the specific angle θ. On the other hand, since we focus on the energy distribution on the θ-dimension in this paper, $\chi (\tau ,f_{d},\theta )$ can be turned into the transmit beampattern by setting τ=0 and $f_{d}=0$, which describes the transmit power distribution in space.

## 3. Ambiguity Function Analysis of the LFM-PC Waveform Set

### 3.1. The Ambiguity Function Derivation

By substituting (1) and (2) into (3), the ambiguity function $\chi (\tau ,f_{d},\theta )$ can be expanded into

(4) $$\begin{array}{cc} \; & \chi \left(\tau ,f_{d},\theta \right)=\int_{-\infty }^{\infty }a_{t}^{T}\left(\theta \right)s\left(t\right)s^{H}\left(t-\tau \right)a_{t}^{*}\left(\theta \right)e^{j2\pi f_{d}t}dt\\ \; & =\;\;\int_{-\infty }^{\infty }\;\;\sum_{m=1}^{M}\;\;u_{m}^{PC}\left(t\right)e^{j\pi \mu t^{2}}e^{j2\pi c_{m}\Delta ft}e^{j2\pi c_{m}d\sin\theta /\lambda }\;\;\sum_{n=1}^{M}\;\;u{{}_{n}^{PC}}^{*}\;\left(t\;-\;\tau \right)e^{-j\pi \mu {\left(t\;-\;\tau \right)}^{2}}\;\;e^{-j2\pi c_{n}\Delta f\left(t\;-\;\tau \right)}\;e^{-j2\pi c_{n}d\sin\theta /\lambda }\;e^{j2\pi f_{d}t}dt\\ \; & =\sum_{m=1}^{M}\sum_{n=1}^{M}e^{j2\pi c_{n}\Delta f\tau }e^{j2\pi \left(c_{m}-c_{n}\right)d\sin\theta /\lambda }\int_{-\infty }^{\infty }u_{m}^{PC}\left(t\right)u{{}_{n}^{PC}}^{*}\left(t-\tau \right)e^{j\pi \mu [t^{2}-\;{\left(t\;-\;\tau \right)}^{2}]}e^{j2\pi \left(c_{m}-c_{n}\right)\Delta ft}e^{j2\pi f_{d}t}dt\\ \; & =e^{-j\pi \mu \tau^{2}}\sum_{m=1}^{M}\sum_{n=1}^{M}e^{j2\pi c_{n}\Delta f\tau }e^{j2\pi \left(c_{m}-c_{n}\right)d\sin\theta /\lambda }\int_{-\infty }^{\infty }u_{m}^{PC}\left(t\right)u{{}_{n}^{PC}}^{*}\left(t-\tau \right)e^{j2\pi \left[\mu \tau +\left(c_{m}-c_{n}\right)\Delta f+f_{d}\right]t}dt, \end{array}$$

where $\mu =\mu_{m}$ is assumed for simplicity. By letting $c_{m}-c_{n}=m-n$, Equation (4) can be further represented as

(5) $$\begin{array}{cc} \; & \chi \left(\tau ,f_{d},\theta \right)\;=\;e^{-j\pi \mu \tau^{2}}\;\;\left[\;\;\;\begin{array}{c} \sum_{m=1}^{M}e^{j2\pi c_{m}\Delta f\tau }\int_{-\infty }^{\infty }u_{m}^{PC}\left(t\right)u{{}_{m}^{PC}}^{*}\left(t\;-\;\tau \right)e^{j2\pi \left(\mu \tau +f_{d}\right)t}dt\\ +\sum_{p=1}^{M-1}\sum_{m=p+1}^{M}e^{j2\pi (c_{m}-p)\Delta f\tau }e^{j2\pi pd\sin\theta /\lambda }\;\;\int_{-\infty }^{\infty }\;u_{m}^{PC}\left(t\right)u{{}_{m-p}^{PC}}^{*}\left(t\;-\;\tau \right)e^{j2\pi \left(\mu \tau +p\Delta f+f_{d}\right)t}dt\\ +\sum_{q=-1}^{-(M-1)}\sum_{m=1}^{M+q}e^{j2\pi (c_{m}-q)\Delta f\tau }e^{j2\pi qd\sin\theta /\lambda }\;\;\int_{-\infty }^{\infty }\;u_{m}^{PC}\left(t\right)u{{}_{m-q}^{PC}}^{*}\left(t\;-\;\tau \right)e^{j2\pi \left(\mu \tau +q\Delta f+f_{d}\right)t}dt \end{array}\;\;\;\right]\\ \; & \overset{▵}{=}\;\;\;\;\begin{array}{c} \sum_{m=1}^{M}\;w_{m,0}\left(\tau ,\theta \right)\chi_{m,0}\left(\tau ,f_{d}\right)\;+\;\sum_{p=1}^{M-1}\sum_{m=p+1}^{M}\;w_{m,p}\left(\tau ,\theta \right)\chi_{m,p}\left(\tau ,f_{d}\right)\;+\;\sum_{q=-1}^{-(M-1)}\sum_{m=1}^{M+q}\;w_{m,q}\left(\tau ,\theta \right)\chi_{m,q}\left(\tau ,f_{d}\right), \end{array} \end{array}$$

where

(6) $$\begin{array}{cc} w_{m,v}\left(\tau ,\theta \right) & =e^{j2\pi (c_{m}-v)\Delta f\tau }e^{j2\pi vd\sin\theta /\lambda }, \end{array}$$

(7) $$\begin{array}{cc} \chi_{m,v}\left(\tau ,f_{d}\right) & =e^{-j\pi \mu \tau^{2}}\int_{-\infty }^{\infty }u_{m}^{PC}\left(t\right)u{{}_{m-v}^{PC}}^{*}\left(t-\tau \right)e^{j2\pi \left(\mu \tau +v\Delta f+f_{d}\right)t}dt, \end{array}$$

and v=m−n∈{−(M−1),⋯,−1,0,1,⋯,(M−1)}. The $\left|\chi_{m,v}\left(\tau ,f_{d}\right)\right|$ is the ambiguity function between the *m*th LFM-PC waveform and the (m−v)th LFM-PC waveform, which can be computed by

(8) $$\begin{array}{cc} \left|\chi_{m,v}\left(\tau ,f_{d}\right)\right| & =\left|\int_{-\infty }^{\infty }s_{m}\left(t\right)s_{m-v}^{*}\left(t-\tau \right)e^{j2\pi f_{d}t}dt\right|\\ \; & =\left|\int_{-\infty }^{\infty }\;\;\;\;u_{m}^{PC}\left(t\right)u{}_{m-v}^{PC*}\left(t\;-\;\tau \;\right)e^{j2\pi \left(\mu \tau +v\Delta f+f_{d}\right)t}dt\right|\\ \; & =\;\;\left|r_{m,m-v}^{PC}\left(\tau \right)\frac{\sin[\pi \left(\mu \tau \;+\;v\Delta f\;+\;f_{d}\right)\left(T_{c}\;-\;\left|\tau \right|\right)]}{\pi \left(\mu \tau \;+\;v\Delta f\;+\;f_{d}\right)\left(T_{c}\;-\;\left|\tau \right|\right)}\left(T_{c}\;-\;\left|\tau \right|\right)\right|. \end{array}$$

The $r_{m,m-v}^{PC}\left(\tau \right)$ is the correlation function of the *m*th phase-coded sequence and the (m−v)th phase-coded sequence. If the phase-coded sequences modulated on different antennas are the same, the $r_{m,m-v}^{PC}\left(\tau \right)$ is irrelevant with *m*, and $\chi_{m,v}\left(\tau ,f_{d}\right)$ becomes $\chi_{v}\left(\tau ,f_{d}\right)$. Then, the ambiguity function $\chi (\tau ,f_{d},\theta )$ and $\left|\chi (\tau ,f_{d},\theta )\right|$ of LFM-PC angular waveforms in (5) become

(9a) $$\begin{array}{c} \begin{array}{cc} \; & \chi \left(\tau ,f_{d},\theta \right)=\;\;\;\;\;\begin{array}{c} \sum_{m=1}^{M}\;W_{0}\left(\tau ,\theta \right)\chi_{0}\left(\tau ,f_{d}\right)\;+\;\sum_{p=1}^{M-1}\;W_{p}\left(\tau ,\theta \right)\chi_{p}\left(\tau ,f_{d}\right)\;+\;\sum_{q=-1}^{-(M-1)}\;W_{q}\left(\tau ,\theta \right)\chi_{q}\left(\tau ,f_{d}\right), \end{array} \end{array} \end{array}$$

(9b) $$\begin{array}{c} \begin{array}{cc} \; & \left|\chi (\tau ,f_{d},\theta )\right|\approx \;\;\;\;\;\begin{array}{c} \sum_{m=1}^{M}\;\left|W_{0}\left(\tau ,\theta \right)\right|\left|\chi_{0}\left(\tau ,f_{d}\right)\right|\;+\;\sum_{p=1}^{M-1}\;\left|W_{p}\left(\tau ,\theta \right)\right|\left|\chi_{p}\left(\tau ,f_{d}\right)\right|\;+\;\sum_{q=-1}^{-(M-1)}\;\left|W_{q}\left(\tau ,\theta \right)\right|\left|\chi_{q}\left(\tau ,f_{d}\right)\right|, \end{array} \end{array} \end{array}$$

where the approximate equaling in (9b) only demonstrates that all the possible peaks of $\left|\chi (\tau ,f_{d},\theta )\right|$ are determined by the sum of products $\left|W_{v}\left(\tau ,\theta \right)\right|\left|\chi_{v}\left(\tau ,f_{d}\right)\right|$, and

(10) $$\begin{array}{cc} \left|W_{v}\left(\tau ,\theta \right)\right| & =\left\{\begin{array}{cc} \left|\sum_{m=v+1}^{M}e^{j2\pi (c_{m}-v)\Delta f\tau }e^{j2\pi vd\sin\theta /\lambda }\right|, & v\geq 0\\ \left|\sum_{m=1}^{M+v}e^{j2\pi (c_{m}-v)\Delta f\tau }e^{j2\pi vd\sin\theta /\lambda }\right|, & v<0 \end{array}\right.\\ \; & =\left|\frac{\sin[\pi \left(M-\left|v\right|\right)\Delta f\tau ]}{\sin(\pi \Delta f\tau )}\right|. \end{array}$$

Equation (9) shows that $\chi (\tau ,f_{d},\theta )$ can be taken as the sum of the products of $W_{v}\left(\tau ,\theta \right)$ and $\chi_{v}\left(\tau ,f_{d}\right)$. The peaks of product $\left|W_{v}\left(\tau ,\theta \right)\right|\left|\chi_{v}\left(\tau ,f_{d}\right)\right|$ in (9b) provide all the possible peaks of $\chi (\tau ,f_{d},\theta )$ on $\left(\tau ,f_{d}\right)$ space. If the peaks of $W_{v}\left(\tau ,\theta \right)\chi_{v}\left(\tau ,f_{d}\right)$ are added in phase for different *v* in (9a), the possible peaks determined by the peaks of $\left|W_{v}\left(\tau ,\theta \right)\right|\left|\chi_{v}\left(\tau ,f_{d}\right)\right|$ will appear for $\chi (\tau ,f_{d},\theta )$. If the peaks of $W_{v}\left(\tau ,\theta \right)\chi_{v}\left(\tau ,f_{d}\right)$ are added in reversed-phase for different *v* in (9a), the possible peaks, determined by the peaks of $\left|W_{v}\left(\tau ,\theta \right)\right|\left|\chi_{v}\left(\tau ,f_{d}\right)\right|$, will disappear for $\chi (\tau ,f_{d},\theta )$. Thus, the ambiguity function behaviour of the LFM-PC angular waveforms is determined by the properties of $\left|W_{v}\left(\tau ,\theta \right)\right|$ and $\left|\chi_{v}\left(\tau ,f_{d}\right)\right|$.

### 3.2. The Preferred PC Sequences

We discuss two cases of the same and different PC sequences modulating on different antennas to provide useful guides on the preferred PC sequences. Combined with the above theoretical derivations, some simulations are conducted with the parameters set in Table 1. The hyper-logistic [18] chaotic phase-coded sequences are utilized in the LFM-PC waveforms. The bandwidths are uniformly equal as $B_{m}=B_{s}$, ∀m. The frequency intervals are uniformly equal as ${\Delta f}_{m}={\Delta f}_{s}=\Delta f$, ∀m.

**Figure 3:**
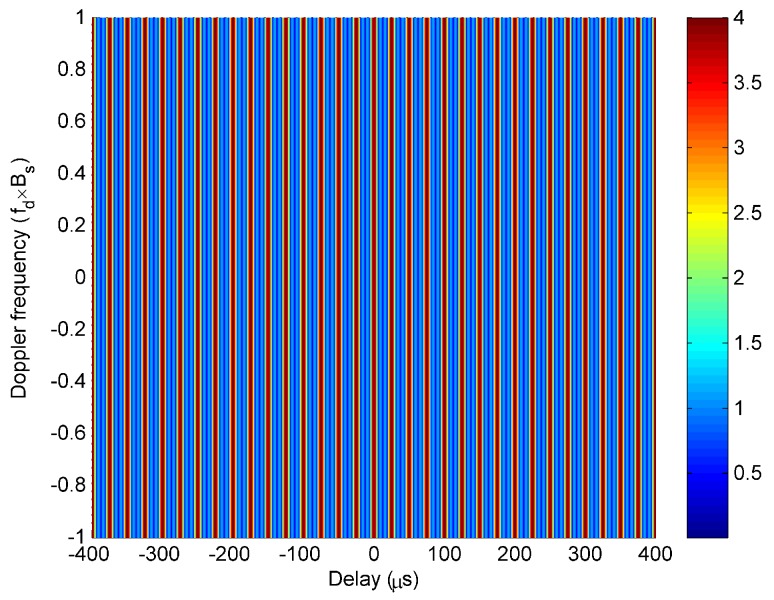
The $\left|W_{v}\left(\tau ,\theta \right)\right|$ for v=3.

First, the case of modulating the same PC sequence is simulated. In this case, the ambiguity function $\left|\chi (\tau ,f_{d},\theta )\right|$ of LFM-PC angular waveforms can be expressed in (9). It is found from (10) that the peaks of $\left|W_{v}\left(\tau ,\theta \right)\right|$ are located at ±nnΔfΔf,θ for $\forall f_{d}$ and n=1,2,⋯,TΔf−1. The peak values of $\left|W_{v}\left(\tau ,\theta \right)\right|$ are the same and equivalent to M−v. The $\left|W_{v}\left(\tau ,\theta \right)\right|$ is a periodic function of τ, and the period is 1/Δf, which does not change with *v*. The $\left|W_{v}\left(\tau ,\theta \right)\right|$ for v=3 is taken as an example and plotted on the $\left(\tau ,f_{d}\right)$ space in Figure 3. The simulation result confirms the theoretical analysis, which shows that the $\left|W_{v}\left(\tau ,\theta \right)\right|$ is the periodic function on the τ-axis, and remains unchanged with $f_{d}$.

**Figure 4:**
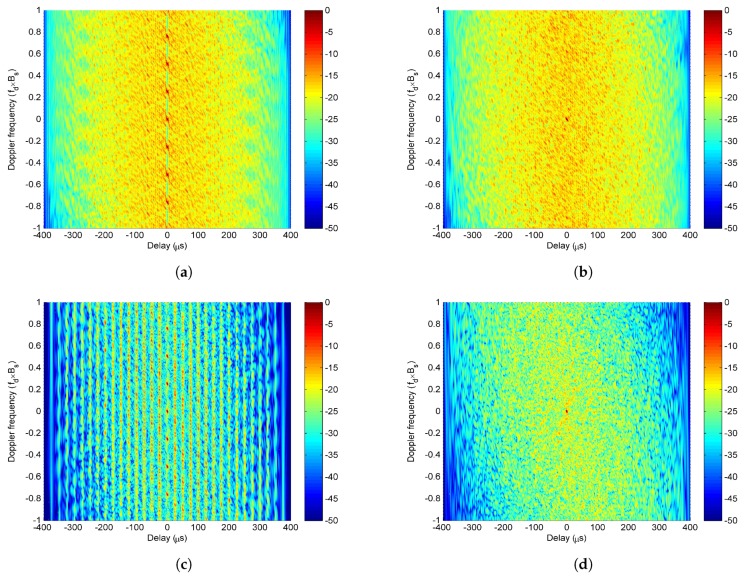
The $\left|\chi_{v}\left(\tau ,f_{d}\right)\right|$ for v=−3,⋯,0,⋯,3 with (**a**) the same modulated sequence; (**b**) different modulated sequences. The ambiguity functions of the LFM-PC waveform set with (**c**) the same modulated sequence; (**d**) different modulated sequences.

It is found from (8) that when v=0, $\left|\chi_{v}\left(\tau ,f_{d}\right)\right|$ denotes the self-ambiguity function of a single LFM-PC waveform, while when v≠0, it denotes the cross-ambiguity function between different LFM-PC waveforms. The cross-ambiguity function can be obtained by translating the self-ambiguity function $\left|\chi_{0}\left(\tau ,f_{d}\right)\right|$ by vΔf along the doppler axis. The peaks of $\left|\chi_{v}\left(\tau ,f_{d}\right)\right|$ are located at $\left(0,-v\Delta f\right)$, and the peak value is $T_{c}\left|r_{m,m-v}^{PC}\left(0\right)\right|$. The $\left|\chi_{v}\left(\tau ,f_{d}\right)\right|$ with the same modulated PC sequence for ν=−3,⋯,0,⋯,3 is plotted in Figure 4a, which is also consistent with the above theoretical analysis. From Figure 4a, we can see that $\left|\chi_{v}\left(\tau ,f_{d}\right)\right|$ has equally-spaced peaks on a zero delay axis, and the distance between adjacent peaks is Δf. When the same phase-coded sequence is modulated on different antennas, the $\left|r_{m,m-v}^{PC}\left(0\right)\right|$ does not change with *v*, then the peak value $T_{c}\left|r_{m,m-v}^{PC}\left(0\right)\right|$ of $\left|\chi_{v}\left(\tau ,f_{d}\right)\right|$ does not change with *v*. Thus, the 2M−1 peaks located at $\left(0,-v\Delta f\right)$ have the same peak value. It is also seen that every single $\left|\chi_{v}\left(\tau ,f_{d}\right)\right|$ has a LFM ridge with a LFM slope, which also shows the delay-doppler coupling.

Next, the case of modulating different PC sequences is simulated. Equation (9) is utilized to analyze the simulation results. Although Equation (9) is deduced by assuming that the same PC sequence is modulated on the LFM-PC waveform set, it is also approximately equivalent to (5) without this assumption. The simulation results do conform with the analysis based on (9). The $\left|\chi_{v}\left(\tau ,f_{d}\right)\right|$ with the different modulated phase-coded sequences for ν=−3,⋯,0,⋯,3 is plotted in Figure 4b. From Figure 4b, we can see that $\left|\chi_{v}\left(\tau ,f_{d}\right)\right|$ has only one peak at the origin of the delay-doppler space, and that LFM ridges are eliminated due to the different sequences. According to Equation (8), when different phase-coded sequences are modulated on different antennas, the cross-correlation function $\left|r_{m,m-v}^{PC}\left(\tau \right)\right|$ is small for probably ∀v≠0 and ∀τ. The peak value $T_{c}\left|r_{m,m-v}^{PC}\left(0\right)\right|$ of $\left|\chi_{v}\left(\tau ,f_{d}\right)\right|$ can be suppressed when v≠0. Then the peak of $\left|\chi_{v}\left(\tau ,f_{d}\right)\right|$ only appears at $\left(0,0\right)$, and other peaks located at $\left(0,-v\Delta f\right)$ for v≠0 are eliminated by the small $\left|r_{m,m-v}^{PC}\left(\tau \right)\right|$.

Finally, the ambiguity functions of the LFM-PC angular waveforms with the same PC sequence and different PC sequences are compared in Figure 4c,d. Based on (9), Figure 4c,d are closely related with the products of Figure 3 and Figure 4a,b. It can be seen from Figure 4c that the ambiguity function of LFM-PC angular waveforms with the same modulated sequence has peaks in delay axis and also doppler axis. This phenomenon is actually caused by the product of $\left|W_{v}\left(\tau ,\theta \right)\right|$ in Figure 3 and $\left|\chi_{v}\left(\tau ,f_{d}\right)\right|$ in Figure 4a. The possible peaks produced by multiplying $\left|W_{v}\left(\tau ,\theta \right)\right|$ in Figure 3 with $\left|\chi_{v}\left(\tau ,f_{d}\right)\right|$ in Figure 4a are widely distributed on $\left(\tau ,f_{d}\right)$ space. The produced peaks will be retained if the peaks of $W_{v}\left(\tau ,\theta \right)\chi_{v}\left(\tau ,f_{d}\right)$ are added in phase for different *v* at a certain $\left(\tau ,f_{d}\right)$ in (9a). The produced peaks will not be retained if the peaks of $W_{v}\left(\tau ,\theta \right)\chi_{v}\left(\tau ,f_{d}\right)$ are added in reversed-phase for different *v* at a certain $\left(\tau ,f_{d}\right)$ in (9a). However, when different modulated sequences are utilized in the LFM-PC waveform set, only one peak arises at the origin of the delay-doppler space in Figure 4d, where the shape of the ambiguity function is a thumbtack. This is as there is only one possible peak produced by multiplying $\left|W_{v}\left(\tau ,\theta \right)\right|$ in Figure 3 with $\left|\chi_{v}\left(\tau ,f_{d}\right)\right|$ in Figure 4b, and this sole peak, which always appears, is located at $\tau =0,f_{d}=0$.

These simulations show that modulating the same PC sequence will cause a serious deterioration on the ambiguity function behaviour of LFM-PC angular waveforms. Thus, we concluded that different phase-coded sequences with good correlation properties are preferred to be modulated in the LFM-PC waveform set for a good ambiguity function.

### 3.3. The Superiority of LFM-PC Waveforms

**Figure 5:**
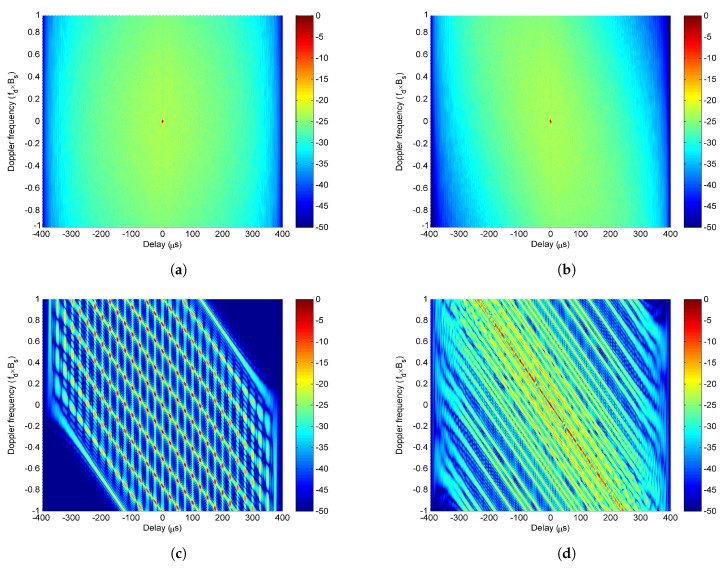
The ambiguity functions of (**a**) orthogonal frequency division (OFD) PC waveform set; (**b**) OFD LFM-PC waveform set; (**c**) OFD LFM waveform set; (**d**) optimized LFM waveform set.

The superiority of the LFM-PC waveform sets over the LFM and PC waveform sets are demonstrated to provide useful guides on designing good LFM-PC waveforms. To compare the ambiguity function of the LFM-PC waveforms with those of other waveforms, different ambiguity functions are plotted in Figure 5 with the simulation parameters in Table 1. We discuss the two kinds of waveform parameters affecting the ambiguity function of LFM-PC waveforms separately. In Figure 5a–c, the effect of modulating PC sequences is individually analyzed by assuming that the frequency intervals and bandwidths are uniform, and ${\Delta f}_{m}=\Delta f$, ∀m. Thus, the waveforms in Figure 5a–c are OFD waveforms. In Figure 5d, the effect of adjusting the frequency intervals and bandwidths is individually analyzed by assuming no PC sequences are modulated.

The average ambiguity functions of the OFD PC and OFD LFM-PC waveform sets with 500 random PC sequence sets are shown in Figure 5a,b. Clearly, the ambiguity function of the OFD PC waveform set in Figure 5a has a thumbtack shape. The ambiguity function of the OFD LFM-PC waveform set in Figure 5b inherits the good properties from the OFD PC waveform set, and also has a thumbtack-shape ambiguity function. However, the ambiguity function of the OFD LFM waveform set in Figure 5c has widely-spread high grating lobes in the delay-Doppler space. These grating lobes can greatly deteriorate the target detection performance. This problem has been pointed in [32,33]; there however, the high grating lobes were only discussed for the autocorrelation function at zero doppler frequency.

In this paper, the high grating lobes are discussed for the ambiguity function, where both zero and nonzero doppler frequencies are considered. Figure 5d gives the ambiguity function of the LFM waveform set proposed in [32]. The LFM waveform set proposed in [32] has non-uniform frequency intervals and bandwidths, which were optimized to eliminate the high grating peaks of the autocorrelation function of the OFD LFM angular waveform. By comparing with Figure 5c, the grating lobes in Figure 5d have greatly reduced, but there are still some residues.

#### 3.3.1. LFM-PC Waveforms vs. PC Waveforms

The ambiguity functions of OFD LFM-PC and OFD PC waveform sets are very similar. When v=0, $\left|\chi_{0}\left(\tau ,f_{d}\right)\right|$ is the self-ambiguity function of the *m*th LFM-PC waveform. $\left|\chi_{0}\left(\tau ,f_{d}\right)\right|$ has an ideal thumbtack shape for a single PC waveform by setting μ=0 in LFM-PC waveform. $\left|\chi_{0}\left(\tau ,f_{d}\right)\right|$ has an approximate thumbtack shape and a less prominent range-doppler coupling for a single LFM-PC waveform. When v≠0, $\left|\chi_{v}\left(\tau ,f_{d}\right)\right|$ is the cross-ambiguity function between the *m*th and the (m−v)th LFM-PC waveforms translated vΔf along the doppler axis, and has no peaks if different sequences are modulated. Therefore, based on above theoretical analysis, both OFD LFM-PC and OFD PC waveform sets have one peak at the origin of range-doppler space. The ambiguity function of the OFD PC waveform set has an ideal thumbtack shape, as shown in Figure 5a. The ambiguity function of the OFD LFM-PC waveform set has an almost thumbtack shape, and the range-doppler coupling is negligible, as shown in Figure 5b.

The obvious advantage for the LFM-PC waveform set is that both the LFM characteristic and PC characteristic are embodied in it, which are reflected from three aspects. First, the range-doppler coupling in the ambiguity function originating from the LFM characteristic has been greatly weakened by the PC characteristic. Secondly, an almost thumbtack shape and low sidelobe levels of the ambiguity function of the LFM-PC waveform set has been strengthened by the PC characteristic. Thirdly, the weak doppler tolerance of PC waveforms is compensated by the strong doppler tolerance of LFM waveforms in the LFM-PC waveforms, thus the sidelobes of the LFM-PC waveforms rise with doppler frequencies less slowly than those of PC waveforms. Accordingly, by formulating an optimization model for LFM-PC waveforms, the LFM characteristic and PC characteristic in it can be balanced and optimized.

#### 3.3.2. LFM-PC Waveforms Vs. LFM Waveforms

The ambiguity function of the OFD LFM waveform set can be derived from that of the LFM-PC waveform set by setting $d_{m,q}=1$ and $c_{m}=m-\left(M+1\right)/2$. For the OFD LFM waveform set, based on (9), $\left|W_{v}\left(\tau ,\theta \right)\right|$ is the same as (10), but $\left|\chi_{v\_\mathrm{OFD}\_\mathrm{LFM}}\left(\tau ,f_{d}\right)\right|$ is rewritten as

(11) $$\begin{array}{cc} \; & \left|\chi_{v\_\mathrm{OFD}\_\mathrm{LFM}}\left(\tau ,f_{d}\right)\right|\;\;=\;\;\;\left|\frac{\sin[\pi \left(\mu \tau \;\;+\;\;v\Delta f\;\;+\;\;f_{d}\right)\left(T_{c}\;\;-\;\;\left|\tau \right|\right)]}{\pi \left(\mu \tau \;\;+\;\;v\Delta f\;\;+\;\;f_{d}\right)\left(T_{c}\;\;-\;\;\left|\tau \right|\right)}\left(T_{c}\;\;-\;\;\left|\tau \right|\right)\right|. \end{array}$$

**Figure 6:**
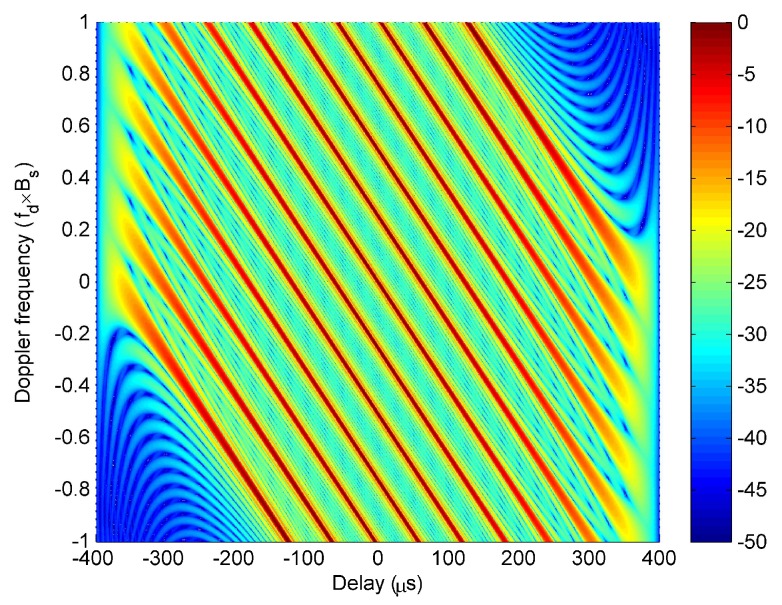
The $\left|\chi_{v}\left(\tau ,f_{d}\right)\right|$ of OFD LFM waveform set for v=−6,⋯,0,⋯,6.

Equation (11) is derived directly from (8) when $\left|r_{m,m-v}^{PC}\left(\tau \right)\right|$ is a constant value for ∀m,v,τ. When v=0, $\left|\chi_{0\_\mathrm{OFD}\_\mathrm{LFM}}\left(\tau ,f_{d}\right)\right|$ represents the ambiguity function of a single LFM, and the shape is an oblique blade. When v≠0, the $\left|\chi_{v\_\mathrm{OFD}\_\mathrm{LFM}}\left(\tau ,f_{d}\right)\right|$ is obtained by translating the $\left|\chi_{0\_\mathrm{OFD}\_\mathrm{LFM}}\left(\tau ,f_{d}\right)\right|$ along the doppler axis. The $\left|\chi_{v\_\mathrm{OFD}\_\mathrm{LFM}}\left(\tau ,f_{d}\right)\right|$ for v=−6,⋯,0,⋯,6 is shown in Figure 6. From Figure 6, the $\left|\chi_{v\_\mathrm{OFD}\_\mathrm{LFM}}\left(\tau ,f_{d}\right)\right|$ for different *v* is the translational version of $\left|\chi_{0\_\mathrm{OFD}\_\mathrm{LFM}}\left(\tau ,f_{d}\right)\right|$ along the doppler axis. Originating from the product of $\left|W_{v}\left(\tau ,\theta \right)\right|$ in Figure 3 and $\left|\chi_{v\_\mathrm{OFD}\_\mathrm{LFM}}\left(\tau ,f_{d}\right)\right|$ in Figure 6, the ambiguity function of OFD LFM angular waveforms in Figure 5c has regularly-distributed peaks in the whole ambiguity function plane. Thus, the superiority of the ambiguity function of LFM-PC waveforms over that of LFM waveforms is obvious.

In [32,33], the grating lobes of the autocorrelation function of the LFM angular waveforms were eliminated by formulating an optimization model with the optimization variables $\Delta f_{m}$ and $B_{m}$. By adjusting the frequency intervals $\Delta f_{m}$ and the bandwidth $B_{m}$ (i.e., the chirp rate, $\mu_{m}$), the properties of $\left|W_{v}\left(\tau ,\theta \right)\right|$ and $\left|\chi_{v\_\mathrm{OFD}\_\mathrm{LFM}}\left(\tau ,f_{d}\right)\right|$ can be changed. The authors in [32,33] only manipulate with the range sidelobes at zero doppler frequency, but we demonstrate in Figure 5d that the grating lobes in the whole ambiguity function plane have been reduced. However, the range-doppler coupling is still prominent, and there are residues of grating lobes, which are those irregular high sidelobes shown in Figure 5d.

Therefore, inspired from the comparison results in Figure 5, we can conclude that: (1) The method of modulating PC sequences is better than the method of adjusting the frequency intervals and bandwidths to solve the grating lobe problem of LFM waveform set. (2) The method of modulating PC sequences on LFM, as shown in Figure 5b, and the method of adjusting the frequency intervals and bandwidths of LFM, as shown in Figure 5d, can be used jointly to optimize a good ambiguity function.

## 4. The Temporal-Spatial Characteristics of LFM-PC Angular Waveforms

To match the desired beampattern and obtain a good ambiguity function, the temporal-spatial characteristics of LFM-PC waveforms should be optimized.

### 4.1. Range Sidelobes of LFM-PC Angular Waveforms

The ambiguity function $\chi (\tau ,f_{d},\theta )$ can be seen as the autocorrelation function of the LFM-PC angular waveforms at the specific doppler frequency $f_{d}$ and spatial angle θ. Thus, the range sidelobe levels at different doppler frequencies are taken as the metric measuring the ambiguity function behaviour. We take $f_{d}=0\;\mathrm{Hz}$ and $\theta =0^{\circ }$ as an example to illustrate the influence of PC sequences, frequency intervals, and bandwidths on the range sidelobes of LFM-PC angular waveforms. A similar analysis and result can also be obtained for LFM-PC angular waveforms at other nonzero doppler frequencies and other directions.

By substituting $f_{d}=0\;\mathrm{Hz}$ and $\theta =0^{\circ }$ into (9), the auto-correlation function of the LFM-PC angular waveforms at zero doppler frequency and zero direction can be obtained, and the $\left|W_{v}\left(\tau ,0\right)\right|$ and $\left|\chi_{m,v}\left(\tau ,0\right)\right|$ become

(12a) $$\begin{array}{cc} \left|W_{v}\left(\tau \right)\right| & =\left|W_{v}\left(\tau ,0\right)\right|=\left|\frac{\sin[\pi \left(M-\left|v\right|\right)\Delta f\tau ]}{\sin(\pi \Delta f\tau )}\right|, \end{array}$$

(12b) $$\begin{array}{cc} \left|\chi_{m,v}\left(\tau \right)\right| & =\left|\chi_{m,v}\left(\tau ,0\right)\right|=\left|r_{m,m-v}^{PC}\left(\tau \right)\frac{\sin[\pi \left(\mu \tau +v\Delta f\right)\left(T_{c}-\left|\tau \right|\right)]}{\pi \left(\mu \tau +v\Delta f\right)\left(T_{c}-\left|\tau \right|\right)}\left(T_{c}-\left|\tau \right|\right)\right|. \end{array}$$

$\left|\chi (\tau ,0,0)\right|$ can be seen as a weighted sum of $\left|\chi_{v}\left(\tau \right)\right|$, and $\left|W_{v}\left(\tau \right)\right|$ is the weighting function. Equation (12) can be compared with (9). Equation (12a) is the same as (10), where the $\left|W_{v}\left(\tau \right)\right|$ is a periodic sampling function with peaks located at $\tau_{1}=\pm n/\Delta f,n=0,1,\cdots ,T\Delta f-1$, and the peak value is M−v. By substituting $f_{d}=0\;\mathrm{Hz}$ into (8), $\left|\chi_{m,v}\left(\tau \right)\right|$ in (12b) is a sinc-like function, and its peak point is located at $\tau_{2}=\frac{-v\Delta f}{\mu }$ and peak value is $\left(T-\left|\tau_{2}\right|\right)r_{m,m-v}^{PC}\left(\tau_{2}\right)$. For OFD LFM waveforms, $r_{m,m-v}^{PC}\left(\tau_{2}\right)$ is a constant, and when the peaks of the sinc-like function $\chi_{v}\left(\tau \right)$ and the weighting function $W_{v}\left(\tau \right)$ approach each other (i.e., $\tau_{1}\approx \tau_{2}$), the sidelobe terms of OFD LFM angular waveforms can be added in phase to cause the discrete grating lobes, which was also illustrated in [32].

To solve the problem of discrete sidelobes, the authors in [32] proposed to adjust the carrier frequency intervals and baseband signal bandwidths of the OFD LFM waveform set to stagger the peak positions of $\left|\chi_{v}\left(\tau \right)\right|$ and $\left|W_{v}\left(\tau \right)\right|$, which makes $\tau_{1}\neq \tau_{2}$. Innovatively, we find that the magnitude of the peak value of $\left|\chi_{m,v}\left(\tau \right)\right|$ for OFD LFM-PC waveform set can be greatly influenced by the peak-to-sidelobe ratio of the correlation function $r_{m,m-v}^{PC}\left(\tau \right)$. Thus, by controlling the modulated phase-coded sequences (i.e., $r_{m,m-v}^{PC}\left(\tau \right)$), the peak values of $\left|\chi_{m,v}\left(\tau \right)\right|$ can be suppressed, and the possible resulting peaks can also be eliminated.

**Figure 7:**
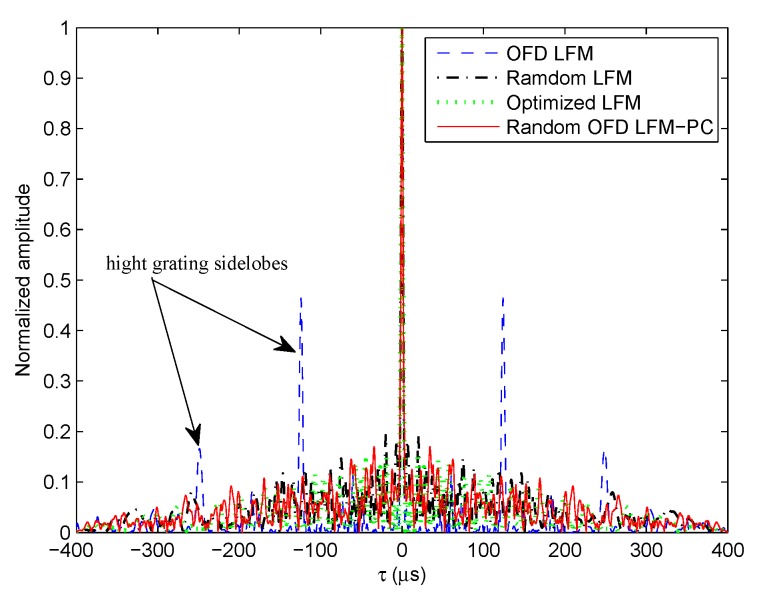
The autocorrelation functions of different angular waveforms.

The auto-correlation sidelobes of OFD LFM angular waveforms, random OFD LFM-PC angular waveforms modulated with random PC sequences, random LFM angular waveforms with random frequency intervals and bandwidths, and the optimized LFM angular waveforms in [32] at $\theta =0^{\circ }$ and $f_{d}=0\;\mathrm{Hz}$ are shown in Figure 7. The simulation parameters are set in Table 1. Different chaotic phase-coded sequences are modulated on different transmit channels. In Figure 7, by comparing the results of the OFD LFM waveform set and the OFD LFM-PC waveform set, it is concluded that the high grating sidelobes of OFD LFM angular waveforms can be greatly suppressed by modulating different phase-coded sequences. By comparing the results of OFD LFM waveform set and the random LFM waveform set, we concluded that the high grating sidelobes of OFD LFM angular waveforms can be suppressed by adjusting the frequency intervals and bandwidths. We also concluded from the result of optimized LFM angular waveforms, in [32], that by optimizing the frequency intervals and bandwidths, the sidelobes can be further reduced.

Thus, by combining the method of the LFM waveform set in [32], which optimizes frequency intervals and carrier bandwidths, and the method of modulating different phase-coded sequences proposed in this paper, an optimization model can be established by jointly optimizing these three variables to suppress range sidelobes of LFM-PC angular waveforms at different Doppler frequencies.

### 4.2. The Transmit Beampattern of LFM-PC Waveforms

When using angular waveforms s(θ,t) as the detection signals, the target detection performance is not only affected by the temporal correlation property of the s(θ,t) but also affected by the energy distribution of the s(θ,t) in the space.

The distribution of electromagnetic energy of LFM-PC waveforms in various directions is reflected by the transmit beampattern. By setting τ=0, and $f_{d}=0$ in (3), the transmit beampattern $P_{E}\left(\theta \right)$ can be written as

(13) $$\begin{array}{cc} P_{E}\left(\theta \right) & =\int_{-\infty }^{\infty }s\left(\theta ,t\right)s^{H}\left(\theta ,t\right)dt=\frac{1}{T}\int_{0}^{T}a_{t}^{T}\left(\theta \right)s\left(t\right)s^{H}\left(t\right)a_{t}^{*}\left(\theta \right)dt\overset{▵}{=}a_{t}^{T}\left(\theta \right){\mathrm{Ra}}_{t}^{*}\left(\theta \right), \end{array}$$

where R is the covariance matrix of the transmitted waveforms, and the element $R_{mi}$ at the *m*th row and the *i*th column of R is

(14) $$\begin{array}{c} R_{mi}=\int_{0}^{T}s_{m}\left(t\right)s_{i}^{*}\left(t\right)dt. \end{array}$$

It is obvious from (13) that the transmit beampattern $P_{E}\left(\theta \right)$ is determined by the waveform covariance matrix R. Thus, in this paper, we approximate the specific desired pattern by optimizing the matrix R.

By substituting (1) into (14) and (13), it shows that frequency intervals, bandwidths and phase-coded sequences of the LFM-PC waveforms all affect the transmit beampattern.

**Figure 8:**
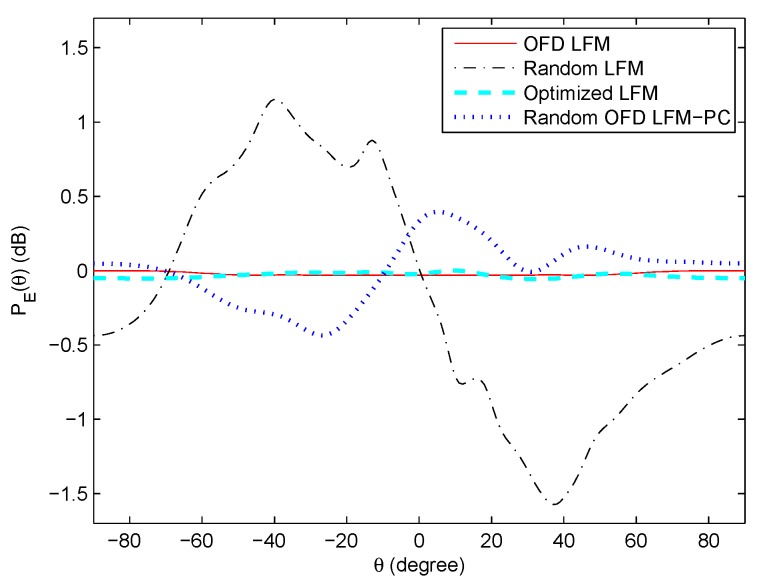
The beampatterns of different angular waveforms.

The transmit beampatterns of the OFD LFM waveform set, random OFD LFM-PC waveform set modulated with random PC sequences, random LFM waveform set with random frequency intervals and bandwiths, and the optimized LFM waveform set proposed in [32] are shown in Figure 8. The beampattern of the OFD LFM is omnidirectional due to the waveform orthogonality; however, the beampatterns of the random OFD LFM-PC and random LFM are not omnidirectional as the phase-coded sequences, the nonuniform frequency intervals, and nonuniform bandwidths affect the waveform correlation property. For the optimized LFM waveform set in [32], a constraint of the omnidirectional transmit beampattern is inserted into the optimization model, thus its transmit beampattern is approximately omnidirectional. Therefore, it is feasible to jointly optimize these three variables of PC sequences, frequency intervals, and bandwidths to approximate the desired beampattern, which is not limited to be omnidirectional.

## 5. Constrained Transmit Beampattern Design

As the frequency intervals, carrier bandwidths, and phase-coded sequences all affect the transmit beampattern and the sidelobe level, we consider optimizing these three variables to approach the desired transmit beampttern constrained by a good ambiguity function behaviour by using the LFM-PC waveforms. The difference between the desired beampattern and the synthesized beampattern and the maximum peak sidelobe level (PSL) of the ambiguity function of angular waveforms within a certain doppler frequency range are taken as the two objective functions. Then, a bi-objective optimization with multiple variables is formulated and solved.

### 5.1. Waveform Covariance Matrix Design

The transmit beampattern design problem can be converted into an optimal design problem of the covariance matrix R of the correlated LFM-PC waveform set. Assume that the entire space $\Theta =[-\frac{\pi }{2},\frac{\pi }{2}]$ is divided into *L* discrete points, and the desired beampattern of MIMO radar is $P_{d}\left(\theta \right),\theta \in \Theta$. Then, the optimization model for the covariance matrix R can be established as

(15) $$\begin{array}{cc} \; & \min_{R,\alpha }\;\;\sum_{l=1}^{L}\left|\alpha P_{d}\left(\theta_{l}\right)-a^{H}\left(\theta_{l}\right)\mathrm{Ra}\left(\theta_{l}\right)\right|\\ \; & s.t.\;\;R\left(m,m\right)=\frac{E}{M},\;m=1,2,\cdots ,M,\\ \; & \;\;\;\;R\geq 0, \end{array}$$

where α is an optimal scaling factor. *E* is the total transmit power, and $\frac{E}{M}$ is the transmit power from each antenna. The objective function is the absolute integral difference between the desired transmit beampattern and actual beampattern. The first constraint means that transmit power from all the antenna elements should be equal, and the second constraint ensures that R is a positive semidefinite matrix. The above optimization problem is convex [38,39], which can be efficiently solved by using the CVX toolbox [40]. The optimal solution of (15) is $R_{d}$.

### 5.2. Optimization Problem Formulation

Based on the waveform covariance matrix design, the formulated problem can be transformed into designing the correlated LFM-PC waveforms for the desired covariance matrix (equivalently the desired transmit beampattern) constrained by the low range sidelobes.

The value of the first objective function corresponding to the beampattern matching error is

(16) $$\begin{array}{c} \mathrm{PAT}:f_{1}\left(\Delta f,\beta_{s},\phi \right)={∥\frac{1}{T}\int_{0}^{T}s\left(t\right)s^{H}\left(t\right)dt-R_{d}∥}_{F}, \end{array}$$

where $R_{d}$ is the desired covariance matrix corresponding to the desired beampattern solved in (15). $\Delta f={\left[\Delta f_{1},\Delta f_{2},\cdots ,\Delta f_{M-1}\right]}^{T}$ is the vector consisting of the frequency intervals, and $c_{m}$ is not constrained to be m−(M+1)/2. $\beta_{s}={\left[B_{1},B_{2},\cdots ,B_{M}\right]}^{T}$ indicates the column vector composed of the bandwidths of transmitted waveforms, where $B_{m}$ is the bandwidth of the *m*th transmitted waveform. $\phi ={\left[\phi_{1,1},\phi_{1,2},\cdots ,\phi_{1,Q},\cdots ,\phi_{M,1},\cdots ,\phi_{M,Q}\right]}^{T}$ is the column vector of phase codes.

The value of the second objective function corresponding to the PSL is

(17) $$\begin{array}{c} \mathrm{PSL}:f_{2}\left(\Delta f,\beta_{s},\phi \right)=\max_{\begin{array}{c} \;0<\tau \leq T\\ k=1,2,3,\cdots ,K\\ l=1,2,3,\cdots ,L \end{array}}\;\left|\chi (\tau ,f_{d}^{k},\theta_{l})\right|, \end{array}$$

where $F_{d}={\left[f_{d}^{1},\cdots ,f_{d}^{k},\cdots ,f_{d}^{K}\right]}^{T}$ is the range of discrete doppler frequencies to be optimized, and $\Theta ={\left[\theta_{1},\cdots ,\theta_{l},\cdots ,\theta_{L}\right]}^{T}$ denotes the discrete search space.

Then, a bi-objective optimization problem for the LFM-PC waveform set is formulated as

(18) $$\begin{array}{cc} \; & P0:\;\min_{\Delta f,\beta_{s},\phi }\;\left\{f_{1}\left(\Delta f,\beta_{s},\phi \right),f_{2}\left(\Delta f,\beta_{s},\phi \right)\right\}\\ \; & s.t.\;\;\sum_{m=1}^{M-1}\Delta f_{m}=B-B_{M},\;\Delta f_{m}>0,\\ \; & \;\;\;\;\frac{1}{T_{c}}\leq B_{i}\leq B-\sum_{m=1}^{i-1}\Delta f_{m},\;i=1,2,\cdots ,M,\\ \; & \;\;\;\;\phi_{m,q}\in \;\;\;\left\{\;\;\;0,\frac{\pi }{2},\pi ,\frac{3\pi }{2}\;\;\;\right\},\;m=1,2,\cdots ,M,\;q=1,2,\cdots ,Q, \end{array}$$

where *B* is the total bandwidth. The first constraint is to ensure that the total bandwidth is unchanged from the perspective of frequency intervals, and that the frequency intervals are positive. The second constraint indicates that the lower limit of the carrier bandwidth is equivalent to the bandwidth of the phase-coded sequence and ensures that the total bandwidth is unchanged from the perspective of the bandwidths. The third constraint indicates that the phase coding is a four-phase coding, which is used for explanation, and other poly-phase codes have similar results. It is found from (18) that by adjusting the parameters $\Delta f,\beta_{s},\phi$, the optimized waveform set can be the LFM-PC, the PC, or the LFM waveform set.

The absolute optimal solution of the bi-objective optimization problem P0 is such that making both PAT and PSL individually reach their minimum. However, the PAT and PSL are mutually restrictive and incompatible. Thus we must explore the optimal solution under certain restrictions or in certain senses.

### 5.3. The Proposed Optimization Algorithm

We propose finding the optimal solution of the bi-objective optimization problem based on two stages of optimization. In the first stage, the PAT and PSL both decrease, and the ε-constraint method [41] in multi-objective optimization theory is utilized. In the second stage, the PAT is taken as the focus of consideration by relaxing the PSL to some extent, and the key objective method [41] in multi-objective optimization theory is exploited.

#### 5.3.1. Stage I-Joint Optimization of the Beampattern and PSL

In the first stage, the beampattern and PSL of the correlated LFM-PC waveforms are jointly optimized. The ε-constraint method puts one objective function in the constraint by giving an upper threshold ε, and then puts the other objective function in the constraint. This process is iterated, and in each iteration, the model of only one objective function with the other objective function as the constraint is built. In this way, the bi-objective optimization problem is transformed into a single-objective optimization problem, iteratively. Each iteration contains two steps as follows.

**Stage I-Step 1**: We first set the maximum PSL as the objective function and set the beampattern matching error as the constraint. Then the first optimization problem is built as

(19) $$\begin{array}{cc} \; & P1(n):\;\min_{\Delta f,\beta_{s}}\;\left\{\max_{\begin{array}{c} \;0<\tau \leq T\\ k=1,2,3,\cdots ,K\\ l=1,2,3,\cdots ,L \end{array}}\;\left|\chi (\tau ,f_{d}^{k},\theta_{l})\right|\;\right\}\\ \; & s.t.\;\;\sum_{m=1}^{M-1}\Delta f_{m}=B-B_{M},\;\Delta f_{m}>0,\\ \; & \;\;\;\;\frac{1}{T_{c}}\leq B_{i}\leq B-\sum_{m=1}^{i-1}\Delta f_{m},\;i=1,2,\cdots ,M,\\ \; & \;\;\;\;{∥\frac{1}{T}\;\;\int_{0}^{T}\;\;\;\;s\left(t\right)s^{H}\left(t\right)dt\;-\;R_{d}∥}_{F}\;\leq \;\varepsilon_{2}\left(n-1\right), \end{array}$$

where P1(n) denotes the optimization problem P1 in the *n*th iteration. The third constraint indicates that the beampattern matching error is bounded by threshold value $\varepsilon_{2}\left(n-1\right)$, which is the optimal beampattern matching error obtained in the second step of stage I at the (n−1)th iteration.

The objective function is not convex. It is a nonlinear programming problem with not only linear equality and inequality constraints, but also nonlinear constraints. As the frequency steps and carrier bandwidths are continuous real variables, the sequence quadratic programming (SQP) algorithm [35] is exploited to solve the problem.

The optimization goal of this step is to further reduce the PSL and reach a local optimum value under the constraint of the matching error value $\varepsilon_{2}\left(n-1\right)$. After solving P1(n), the optimum value of the objective function is

(20) $$\varepsilon_{1}\left(n\right)={\left\{\max_{\begin{array}{c} \;0<\tau \leq T\\ k=1,2,3,\cdots ,K\\ l=1,2,3,\cdots ,L \end{array}}\;\left|\chi (\tau ,f_{d}^{k},\theta_{l})\right|\right\}}_{n}.$$

**Stage I-Step 2**: We set the beampattern matching error as the objective function and set maximum PSL as the constraint. The second optimization model is established as

(21) $$\begin{array}{cc} \; & P2\left(n\right):\;\min_{\phi }\;\left\{{∥\frac{1}{T}\;\;\int_{0}^{T}\;\;\;\;\;s\left(t\right)s^{H}\left(t\right)dt\;-\;R_{d}∥}_{F}\;\;\;\;\right\}\\ \; & s.t.\;\;\;\phi_{m,q}\in \;\;\;\left\{\;\;\;0,\frac{\pi }{2},\pi ,\frac{3\pi }{2}\;\;\;\right\},\;m=1,2,\cdots ,M,\;q=1,2,\cdots ,Q,\\ \; & \;\;\;\;\;\max_{\begin{array}{c} \;0<\tau \leq T\\ k=1,2,3,\cdots ,K\\ l=1,2,3,\cdots ,L \end{array}}\;\left|\chi (\tau ,f_{d}^{k},\theta_{l})\right|\;\leq \varepsilon_{1}\left(n\right), \end{array}$$

where P2(n) denotes the optimization problem P2 in the *n*th iteration. The second constraint condition indicates that the maximum PSL is bounded by the threshold value $\varepsilon_{1}\left(n\right)$, which is the optimal PSL obtained in the first step of stage I at the *n*th iteration.

We utilize the adaptive clonal selection (ACS) algorithm [36] as the optimization algorithm, and the optimization goal of this step is to further reduce the beampattern matching error and achieve a local optimum value under the PSL constraint value $\varepsilon_{1}\left(n\right)$. After solving P2(n), the optimum value of the objective function is

(22) $$\varepsilon_{2}\left(n\right)={\left\{{∥\frac{1}{T}\int_{0}^{T}s\left(t\right)s^{H}\left(t\right)dt-R_{d}∥}_{F}\right\}}_{n}.$$

It is clear that the Δf and $\beta_{s}$ are continuous real variables, and ϕ consists of discrete real variables. Thus, we optimize these two groups of variables alternatively in two steps as the alternative optimization method in [42]. Each solution in iteration can be called a weakly valid solution in multi-objective optimization. Therefore, multiple iterations are needed to reduce the values of the two objectives so as to achieve a relatively effective solution.

The initial parameters of each iteration are the optimized parameters of the last iteration. We stop iterating when both the PAT and the maximum PSL no longer decrease. The designed LFM-PC waveforms at the iteration termination point can be a valid solution for the optimization problem in this paper.

#### 5.3.2. Stage II-Mandatory Optimization of the Beampattern

After the first stage of joint optimization of the beampattern and PSL, the two objectives can not decrease simultaneously. However, we can further decrease one objective by relaxing the other objective through optimization.

Therefore, on the basis of the valid solution obtained in Stage I above, the idea of the key objective method in multi-objective optimization theory is exploited, where the beampattern matching error is regarded as the key objective to be mandatorily constrained. Then, we can obtain the optimal synthesized transmit beampattern and relatively superior PSL.

The iterative optimization of the key objective method is conducted. As the optimization variables include the continuous and discrete ones, there are also two steps in each iteration as follows.

**Stage II-Step 1**: The maximum PSL is set as the objective function, and the transmit beampattern matching error is set as the constraint under a threshold. Then the third optimization problem is established as

(23) $$\begin{array}{cc} \; & P3(n):\;\min_{\Delta f,\beta_{s}}\;\left\{\max_{\begin{array}{c} \;0<\tau \leq T\\ k=1,2,3,\cdots ,K\\ l=1,2,3,\cdots ,L \end{array}}\;\left|\chi (\tau ,f_{d}^{k},\theta_{l})\right|\;\right\}\\ \; & s.t.\;\;\sum_{m=1}^{M-1}\Delta f_{m}=B-B_{M},\;\Delta f_{m}>0,\\ \; & \;\;\;\;\frac{1}{T_{c}}\leq B_{i}\leq B-\sum_{m=1}^{i-1}\Delta f_{m},\;i=1,2,\cdots ,M,\\ \; & \;\;\;\;{∥\frac{1}{T}\;\;\int_{0}^{T}\;\;\;\;s\left(t\right)s^{H}\left(t\right)dt\;-\;R_{d}∥}_{F}\;\leq \;\varepsilon_{4}\left(n-1\right), \end{array}$$

where P3(n) denotes the optimization problem P3 in the *n*th iteration. The threshold $\varepsilon_{4}\left(n-1\right)$ is the beampattern matching error obtained in the second step of Stage II at the (n−1)th iteration. The SQP algorithm is utilized to solve P3(n). After solving P3(n), the optimum value of the beampattern matching error is

(24) $$\varepsilon_{3}\left(n\right)={\left\{{∥\frac{1}{T}\int_{0}^{T}s\left(t\right)s^{H}\left(t\right)dt-R_{d}∥}_{F}\right\}}_{n}.$$

**Stage II-Step 2**: The maximum PSL is set as the objective function, and the transmit beampattern matching error is set as the constraint under a lower threshold. Then the fourth optimization problem is established as

(25) $$\begin{array}{cc} \; & P4(n):\;\min_{\phi }\;\left\{\max_{\begin{array}{c} \;0<\tau \leq T\\ k=1,2,3,\cdots ,K\\ l=1,2,3,\cdots ,L \end{array}}\;\left|\chi (\tau ,f_{d}^{k},\theta_{l})\right|\;\right\}\\ \; & s.t.\;\;\phi_{m,q}\in \;\;\;\left\{\;\;\;0,\frac{\pi }{2},\pi ,\frac{3\pi }{2}\;\;\;\right\},\;m=1,2,\cdots ,M,\;q=1,2,\cdots ,Q,\\ \; & \;\;\;\;{∥\frac{1}{T}\;\;\int_{0}^{T}\;\;\;\;s\left(t\right)s^{H}\left(t\right)dt\;-\;R_{d}∥}_{F}\;\leq \;\varepsilon_{3}\left(n\right), \end{array}$$

where P4(n) denotes the optimization problem P4 in the *n*th iteration. The threshold $\varepsilon_{3}\left(n\right)$ is the beampattern matching error obtained in the first step of Stage II at the *n*th iteration. The ACS algorithm is utilized to solve P4(n). After solving P4(n), the optimum value of the beampattern matching error is

(26) $$\varepsilon_{4}\left(n\right)={\left\{{∥\frac{1}{T}\int_{0}^{T}s\left(t\right)s^{H}\left(t\right)dt-R_{d}∥}_{F}\right\}}_{n}.$$

The initial parameters of each iteration are the optimized parameters of the last iteration. It is clear that $\cdots \geq \varepsilon_{3}\left(n-1\right)\geq \varepsilon_{4}\left(n-1\right)\geq \varepsilon_{3}\left(n\right)\geq \varepsilon_{4}\left(n\right)\geq \cdots$. After iteratively optimizing the problems P3(n) and P4(n), the beampattern matching error becomes lower and lower. The beampattern matching error is mandatorily reduced, while the maximum PSL is relaxed. However, the PSL is still acceptable due to the first stage of optimization. We determine the solution obtained at this stage as the optimal solution of the constrained beampattern design problem in this paper.

### 5.4. Detailed Execution and Complexity Analysis

#### 5.4.1. Detailed Execution of the Optimization Algorithm

Combining the joint optimization of Stage I and the mandatory optimization of Stage II, the basic steps of the total optimization process above are summarized as follows.

1. According to the desired beampattern, $P_{d}\left(\theta \right)$, the desired waveform covariance matrix, $R_{d}$, is obtained by solving the convex optimization in (15).
2. The initial frequency intervals Δf, carrier bandwidths $\beta_{s}$ and phase codes ϕ of the correlated LFM-PC waveforms are randomly selected under their corresponding constraints. The initial phase codes ϕ are generated from the hyper-logistic chaotic sequences.
3. We generate an initial threshold value, $\varepsilon_{2}\left(0\right)=\varepsilon_{0}$, to start the optimization problem P1(n) (i.e., the Stage I of optimization). We use the optimum value $\varepsilon_{2}\left(n-1\right)$ of the objective function in P2(n−1) as the constraint threshold in P1(n), and solve the optimization problem, P1(n), by the SQP algorithm. Then, we use the optimum value, $\varepsilon_{1}\left(n\right)$, of the objective function in P1(n) as the constraint threshold in P2(n), and solve the optimization problem, P2(n), with the ACS algorithm. Iterating Stage I-Step 1 and Stage I-Step 2 as ⋯→P1(n−1)→P2(n−1)→P1(n)→P2(n)→⋯. The iterations in the first stage terminate when both the PAT and the maximum PSL no longer decrease.
4. We utilize the optimal parameters at the end of Stage I as the initial parameters of Stage II. We use the optimal value $\varepsilon_{4}\left(n-1\right)$ of the beampattern matching error in P4(n−1) as the constraint threshold in P3(n), and solve the optimization problem, P3(n), by SQP algorithm. Then, we use the optimal value, $\varepsilon_{3}\left(n\right)$, of the beampattern matching error in P3(n) as the constraint threshold in P4(n), and solve the optimization problem, P4(n), by ACS algorithm. Iterating Stage II-Step 1 and Stage II-Step 2 of the second stage as ⋯→P3(n−1)→P4(n−1)→P3(n)→P4(n)→⋯. The iterations terminate when the PAT no longer decreases or the iteration number reaches the maximum number $I_{m}$.

**Figure 9:**
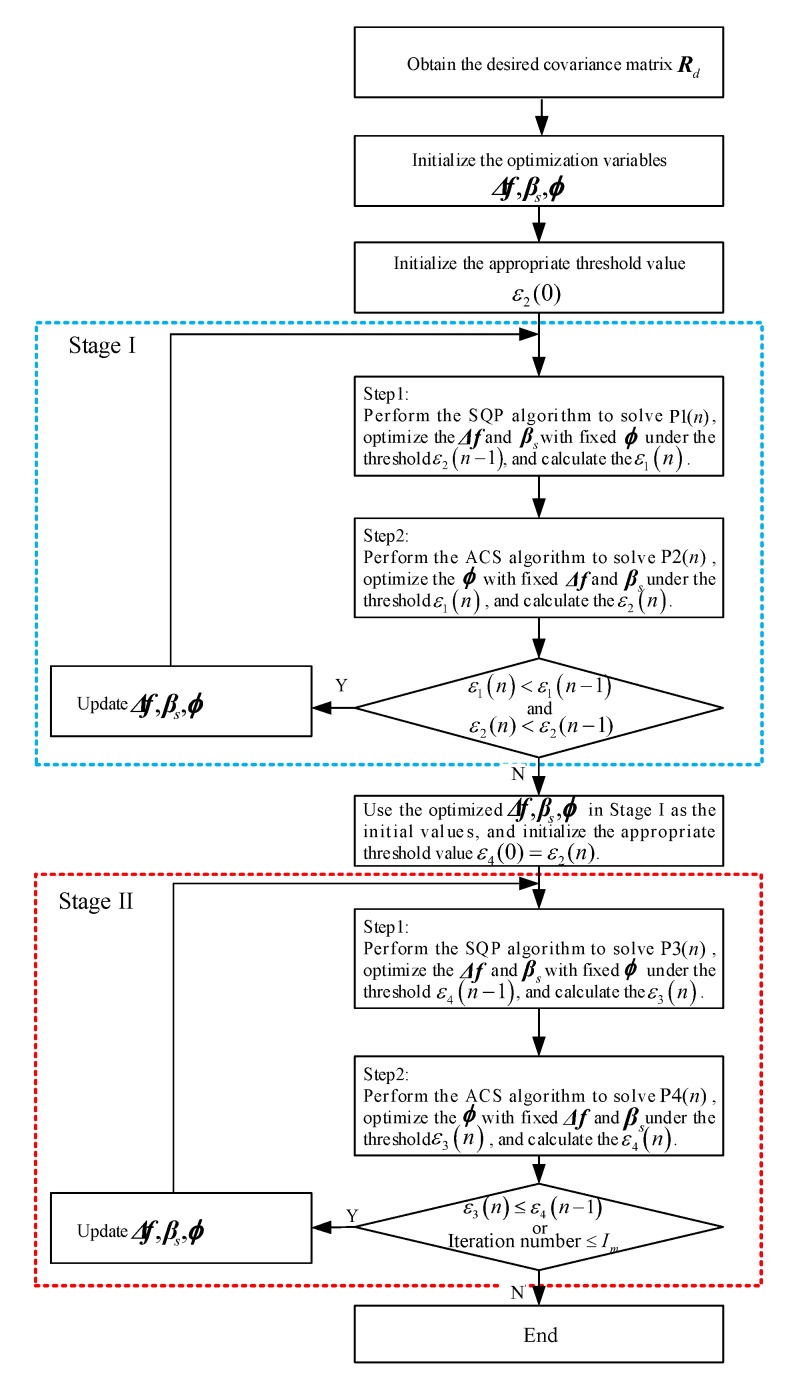
The flowchart of proposed optimization algorithm.

The flowchart for the overall optimization algorithm is shown in Figure 9.

#### 5.4.2. Complexity Analysis of the Optimization Algorithm

The complexity of the main steps in our optimization algorithm is analyzed as follows.

(1) The complexity of SQP: The implementation of the SQP algorithm consists of three main stages, which are updating the Hessian matrix, the quadratic programming solution, and line search, and the merit function [43]. Among these stages, the most costly step should be either the Hessian updating or the QR factorization. For an SQP problem with *X* variables and *Y* constraints, the complexity of Hessian matrix updating is $O\left(X^{2}\right)$, and the complexity of QR factorization is $O\left(Y^{2}X\right)$. The overall complexity is $O\left(X^{2}+Y^{2}X\right)$.

(2) The complexity of calculating the maximum PSL in (17): The ambiguity function at each doppler frequency and space angle can be regarded as a correlation function of the spatial synthesized signal. For *M* transmit antennas, the complexity of calculating the spatial synthesized signal with Q length is $O\left(MQ\right)$. For a *Q* length of spatial synthesized waveform sequence, the correlation function is calculated by the Fast-Fourier-Transform (FFT) for the saving computation, and the complexity is approximately $O\left(Q\log_{2}Q\right)$. These two main steps are repeated for *K* doppler frequencies and *L* spatial angles. Thus, the overall complexity for calculating the maximum PSL is $O\left(KLMQ+KLQ\log_{2}Q\right)$.

(3) The complexity of calculating the beampattern matching error in (16): For a waveform set of waveform number *M* and waveform length *Q*, the complexity for calculating the waveform covariance matrix $R\overset{▵}{=}\frac{1}{T}\int_{0}^{T}s\left(t\right)s^{H}\left(t\right)dt$ is $O\left(M^{2}Q\right)$. The complexity for calculating the norm error of ${∥R-R_{d}∥}_{F}$ is $O\left(M^{3}\right)$. Thus the overall complexity for calculating the PAT is $O\left(M^{2}Q+M^{3}\right)$.

(4) The complexity of ACS algorithm: The ACS algorithm consists of seven steps of population initialization, affinities calculation and ordering, clonal operation, mutation operation, clone selection operation, population regeneration, and termination. We assume the population number of antibodies is $N_{p}$. After cloning and mutation, the population number is changed into $N_{pq}$. To find the best antibody, the affinities of all the antibodies in the population (i.e., the objective function) have to be calculated. Thus in one iteration of the ACS algorithm, the complexity is about $O\left(N_{pq}T_{o}\right)$, where $T_{o}$ is the complexity of computing the affinity function. If the iteration number of ACS is $I_{acs}$, the overall complexity of ACS is $O\left(I_{acs}N_{pq}T_{o}\right)$.

Finally, according to the detailed execution of the optimization algorithm in Section 5.4.1, the overall computation complexity *C* of our proposed algorithm is

(27) $$\begin{array}{rr} \;C= & O\left(2I_{m}\left[{\left(2M\right)}^{2}+{\left(2M+1\right)}^{2}2M\right]\right)+O\left(2I_{m}\left[KLMQ+KLQ\log_{2}Q\right]\right)+O\left(2I_{m}\left[M^{2}Q+M^{3}\right]\right)\\ \; & \;\;+O\left(2I_{m}I_{acs}N_{pq}\left[\left(KLMQ+KLQ\log_{2}Q\right)+\left(M^{2}Q+M^{3}\right)\right]\right), \end{array}$$

where $I_{m}$ is the maximum iteration number, and $O\left(\cdot \right)$ is the big-O notation.

## 6. Simulation Analysis

In this section, simulations are provided to verify the efficiency of the proposed algorithm. A colocated MIMO radar with a ULA is considered. The number of transmit antennas is M=7, and the pulse duration is T=400us. The length of the phase-coded sequence is Q=63, and the total bandwidth is B=500kHz. The initial frequency intervals and carrier bandwidths of the LFM-PC waveforms are randomly selected under their corresponding constraints. The initial phase codes are chosen from the hyper-logistic chaotic sequence, and difference sequences are modulated on different carriers. The discrete space search range is assumed to be $\Theta =[-90^{\circ },\cdots ,-1^{\circ },0^{\circ },1^{\circ },\cdots ,90^{\circ }]$ with a sampling grid of $1^{\circ }$. The discrete doppler frequency range is assumed to be $F_{d}={\left[-0.1/T_{c},\cdots ,-0.004/T_{c},0,0.004/T_{c},\cdots ,0.1/T_{c}\right]}^{T}$ with a sampling grid of $0.004/T_{c}$.

### 6.1. One-Main-Lobe Scenario

We first focus on a one-main-lobe scenario. A symmetric beampattern of mainlobe width $60^{\circ }$ is desired, where the mainlobe region $\left[-30^{\circ },30^{\circ }\right]$ and the sidelobe regions $\left[-90^{\circ },-30^{\circ }\right]\cup \left[30^{\circ },90^{\circ }\right]$ are both uniformly discretized with a grid size $1^{\circ }$. In this case, the desired beampattern can be represented as $P_{d}\left(\theta \right)=\left\{\begin{array}{cc} 1, & \theta \in [-30^{\circ },30^{\circ }]\\ 0, & \mathrm{otherwise} \end{array}\right.$.

**Figure 10:**
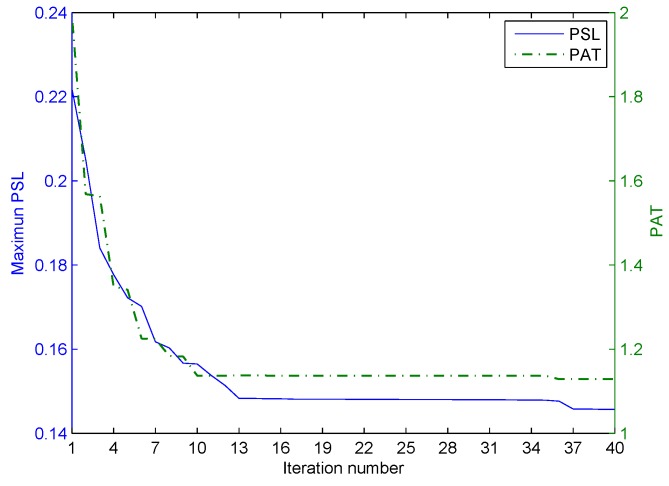
The iterative process of optimization in the first stage for the one-main-lobe.

**Figure 11:**
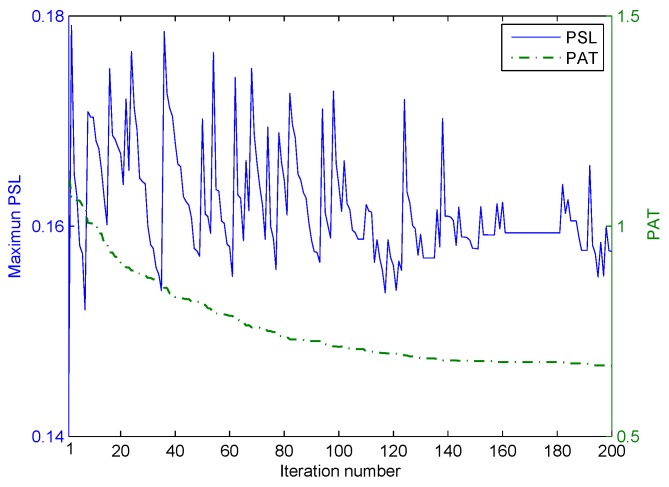
The iterative process of optimization in the second stage for the one-main-lobe.

The first stage of the iterative optimization is carried out for the correlated LFM-PC waveform set first. The values of the two objective functions, PSL and PAT, varied with the iteration number at Stage I of the optimization, are shown in Figure 10. In Figure 10, both the PSL of angular waveforms and the PAT are reduced with the iteration number. The ε-constraint method with one objective function alternating as the objective and the other objective function alternating as the constraint can effectively accelerate the convergence speed. Then, the second stage of iterative optimization is performed by setting the beampattern matching error as the key objective. The values of the two objective functions, PSL and PAT, varied with the iteration number at Stage II of optimization are shown in Figure 11. In Figure 11, the beampattern matching error is further reduced; however, the peak sidelobe level will jitter. This phenomenon is consistent with the concept there is no absolute optimal solution in the two objectives optimization problem.

By the proposed optimization algorithm, the optimized bandwidths, frequency intervals, and phase-coded sequences of seven optimized waveforms are provided in Table 2 and Table 3 respectively, where the optimized waveform data after both Stage I and Stage II is given, and **$W_{1}$**–**$W_{7}$** index the seven waveforms.

**Figure 12:**
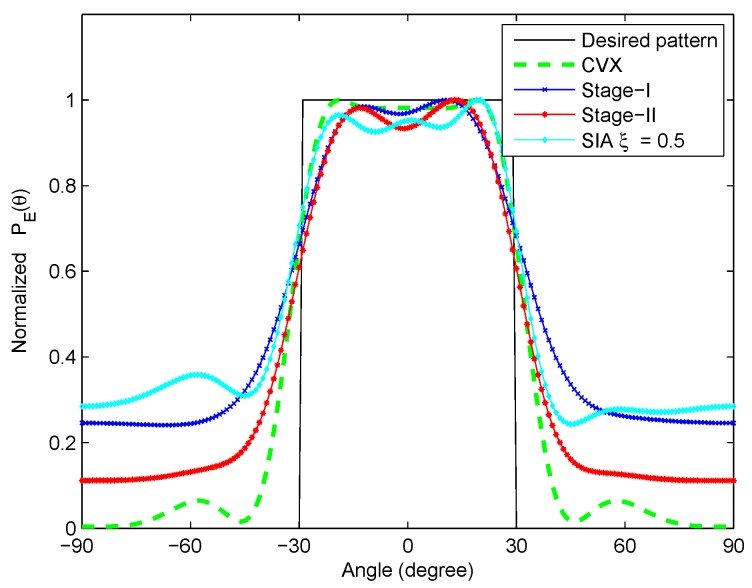
The optimized beampatterns for the one-main-lobe.

By substituting the optimal waveform parameters obtained at the end of Stage I and Stage II of optimization into the correlated LFM-PC waveform set respectively, we can obtain the transmit beampatterns of the correlated LFM-PC waveforms at Stage I and Stage II in Figure 12. As shown in Figure 12, the beampattern designed by CVX is the benchmark, and the synthesized beampatterns at both Stage I and Stage II of optimization approach the desired beampattern well. Compared with the synthesized beampattern at Stage I, the synthesized beampattern at Stage II is more closely approaching the benchmark. The transmit beampattern of our proposed algorithm is also compared with that of the sequential iterative algorithm (SIA) proposed in [28] with ξ=0.5, where the waveform parameters are the same as those in this paper, and the LFM reference waveforms given in [28] are utilized. It is observed from Figure 12 that our proposed algorithm can obtain better transmit beampattern than the SIA to match the desired beampattern.

**Figure 13:**
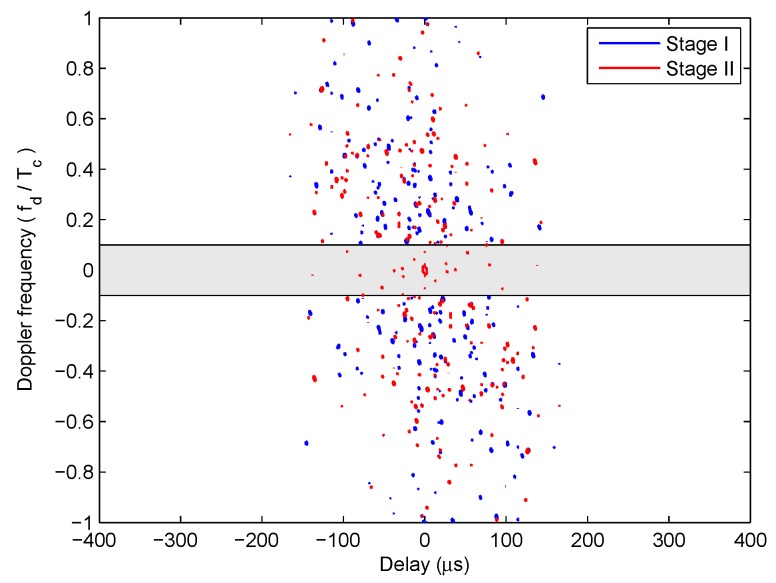
The sidelobes of the optimized LFM-PC waveforms for the one-main-lobe.

The −16.5 dB contour maps of ambiguity functions of the optimized LFM-PC angular waveforms at Stage I and Stage II are both shown in Figure 13. The area of grey shadow in Figure 13 denotes the optimized Doppler frequency range $F_{d}$. This range is not wide due to the heavy computation load. It is shown in Figure 13 that the sidelobes of optimized LFM-PC angular waveforms within the Doppler frequency range $F_{d}$ at the end of Stage I have been reduced and are below -16.5 dB. After the Stage II of optimization, the sidelobes of optimized LFM-PC angular waveforms within the Doppler frequency range $F_{d}$ increase a little bit and are slightly above −16.5 dB.

Figure 12 and Figure 13 demonstrate that the termination point of the first stage of optimization can provide a valid solution, and the beampattern and range sidelobes of LFM-PC waveforms are acceptable; however, they can be further optimized. In the second stage of iterative optimization, the transmit beampattern is mandatorily optimized. The final optimized waveform parameters of Stage II of the iterative optimization are taken as the optimal solution of the constrained beampattern design problem in this paper.

**Figure 14:**
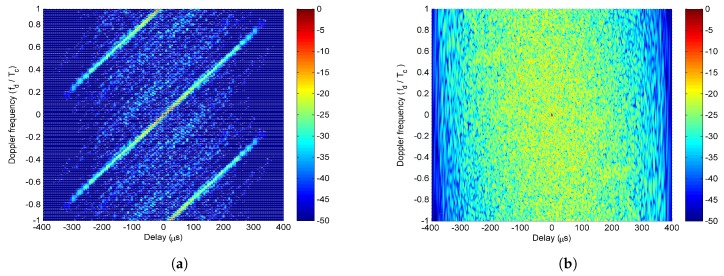
For one-main-lobe, the ambiguity functions of (**a**) the approximate linear-frequency-modulation (LFM) waveforms by sequential iterative algorithm (SIA), ξ=0.5; (**b**) the optimized LFM-PC waveforms.

The ambiguity functions of the approximate LFM waveforms designed by SIA with ξ=0.5 and the LFM-PC waveforms after two stages of our proposed optimization algorithm are shown in Figure 14a,b respectively. Obviously, the waveforms optimized by our proposed algorithm have a thumbtack-shape ambiguity function, while the approximate LFM waveforms designed by SIA have a worse ambiguity function with a serous delay-Doppler coupling. Moreover, by comparing with the ambiguity function in Figure 5c,d, the ambiguity function of the optimized LFM-PC waveforms is much better, and at the same time, the desired beampattern can be approached. The sidelobes within the Doppler frequency range $F_{d}$ have been suppressed, but the sidelobes outside $F_{d}$ are not suppressed. The more Doppler frequencies that are considered, the heavier the computation load is.

For the one-main-lobe scenario, Table 4 further verifies the efficiency of the proposed optimization in the temporal and spatial characteristics of the correlated LFM-PC waveforms, quantitatively. We conclude from Table 4 that: (1) The range sidelobes of LFM-PC waveforms before or after optimization are much lower than those of the OFD LFM waveforms. (2) The OFD LFM waveforms and the correlated LFM-PC waveforms before optimization cannot approach the desired one-main-lobe beampattern; however, the LFM-PC waveforms after optimization can match the desired beampattern well, and the matching error is small. (3) The real values of the PSL drop from 0.2218 before optimization to 0.1576 after two stages of optimization, and the corresponding dB values drop from −13.8080 dB before optimization to -16.0489 dB after two stages of optimization. (4) The beampattern matching errors drop from 1.9919 before optimization to 0.6677 after two stages of optimization. (5) From the joint optimization at Stage I to the mandatory optimization at Stage II, the matching error is reduced, but the sidelobes have slightly increased. The mandatory optimization of the beampattern is achieved at the slight loss of the sidelobe performance. (6) Our proposed waveforms (random LFM-PC or optimized LFM-PC) outperform the SIA-designed waveforms in [28] in terms of both the PSL and PAT performance.

### 6.2. Two-Main-Lobe Scenario

**Figure 15:**
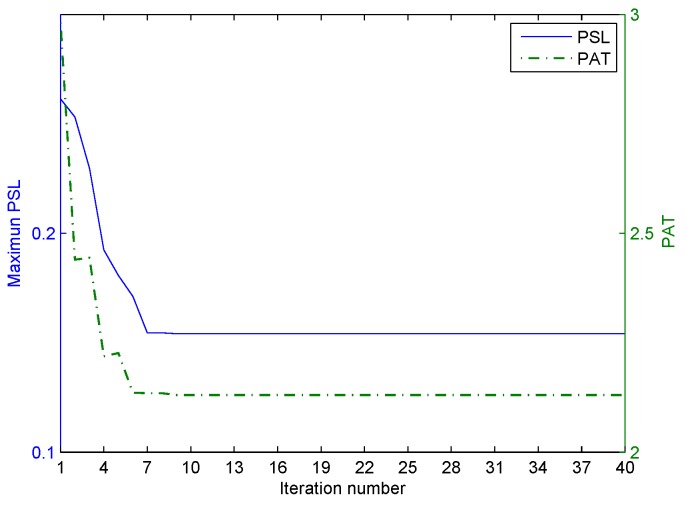
The iterative process of optimization in the first stage for the two-main-lobe.

**Figure 16:**
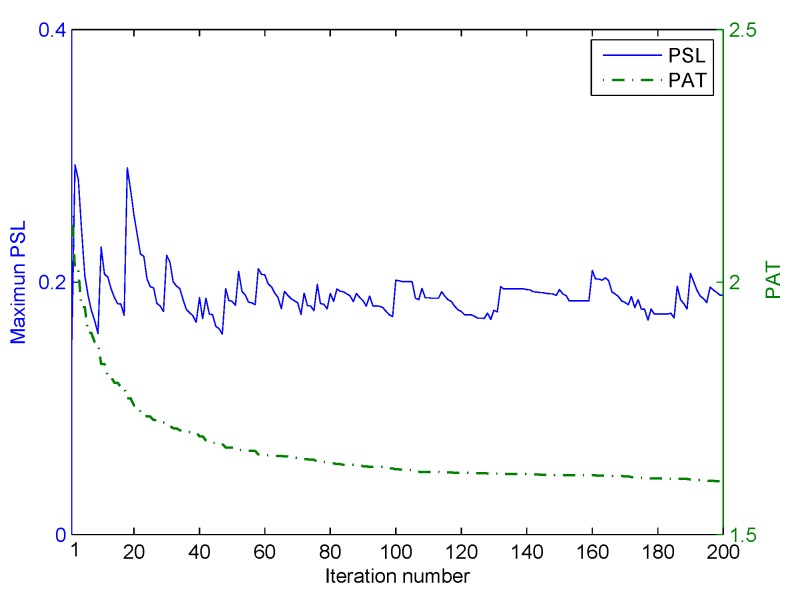
The iterative process of optimization in the second stage for the two-main-lobe.

We consider a two-main-lobe scenario, where the desired beampattern has two beams directed at $-40^{\circ }$ and $40^{\circ }$, and the mainlobe width is $40^{\circ }$. Then, the desired pattern can be represented as $P_{d}\left(\theta \right)=\left\{\begin{array}{cc} 1, & \theta \in \left[-60^{\circ },-20^{\circ }\right]\cup \left[20^{\circ },60^{\circ }\right]\\ 0, & \mathrm{otherwise} \end{array}\right.$, and the discrete grid is $1^{\circ }$. Similarly, the first stage of the iterative optimization is first performed, and the values of the two objective functions, PSL and PAT, varied with the iteration number at Stage I of optimization, are shown in Figure 15. As shown in Figure 15, both the PSL and PAT have decreased. Then, the second stage of iterative optimization is performed by setting the beampattern matching error as the key objective. The values of two objective functions PSL and PAT, varied with the iteration number at Stage II of optimization, are shown in Figure 16. As shown in Figure 16, the PSL of optimized angular waveforms has a small rise, but the PAT of optimized angular waveforms has decreased considerably.

Similarly, the optimized bandwidths, frequency intervals, and phase-coded sequences of seven optimized waveforms after Stage I and Stage II are summarized in Table 5 and Table 6 respectively.

**Figure 17:**
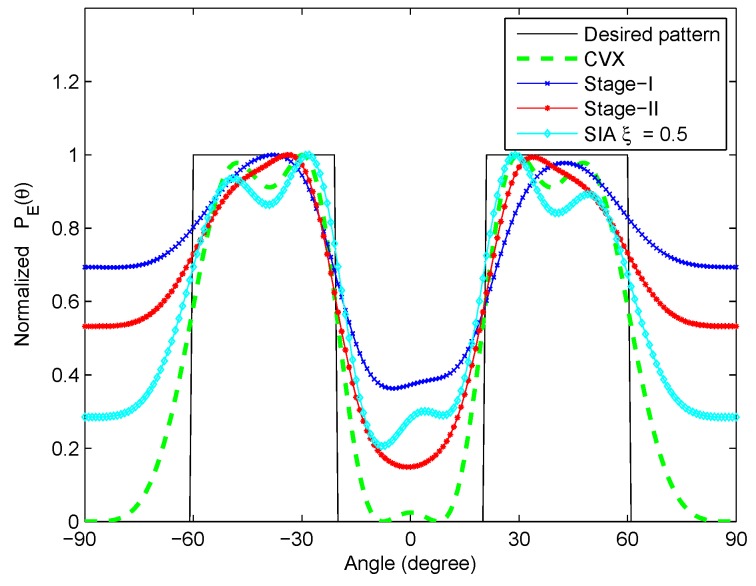
The optimized beampatterns for the two-main-lobe.

**Figure 18:**
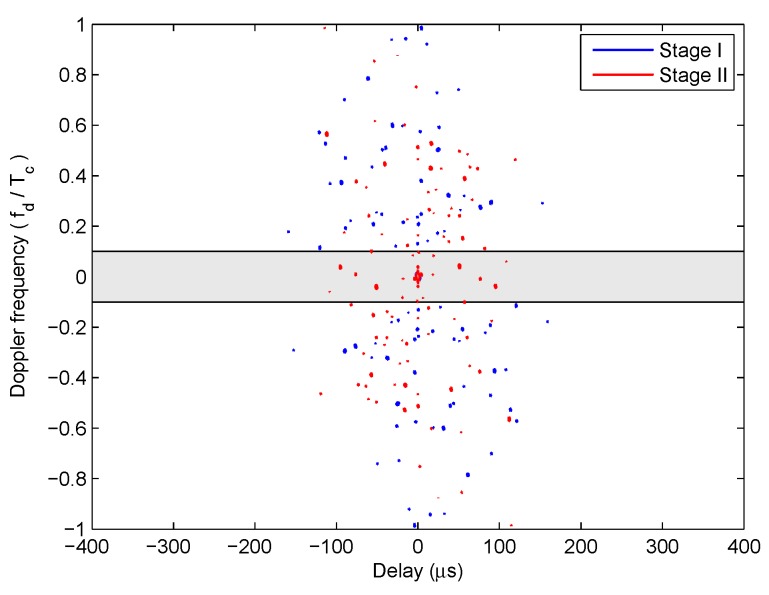
The sidelobes of the optimized LFM-PC waveforms for the two-main-lobe.

By substituting the waveform parameters obtained at the termination points of Stage I and Stage II of optimization into the correlated LFM-PC waveforms, the transmit beampatterns are shown in Figure 17. The −16.28 dB contour maps of ambiguity functions are shown in Figure 18. The optimization results are similar with those in the scenario of one-main-lobe. The beampattern designed via CVX is the benchmark. The beampattern designed by the SIA in [28] is also utilized as a comparison method. It is demonstrated that our synthesized transmit beampattern approaches the desired beampattern well, and the PSL of angular waveforms within the predetermined doppler frequency range $F_{d}$ reaches a low level at the first stage of optimization, which is below −16.28 dB. By the second stage of the optimization, our synthesized beampattern is much better in terms of approaching the desired beampattern, while the PSL within $F_{d}$ rises a little bit, and is above −16.28 dB. The rises of the PSL is negligible, and the transmit beampattern is mandatorily optimized.

**Figure 19:**
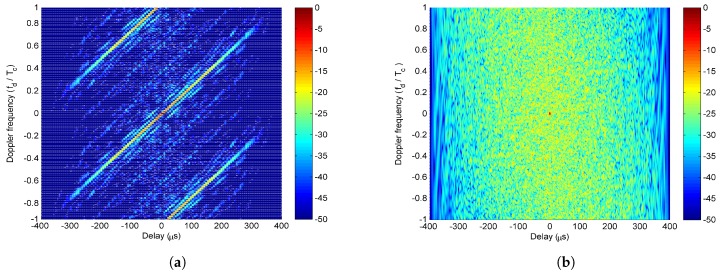
For the two-main-lobe, the ambiguity functions of (**a**) the approximate LFM waveforms by SIA, ξ=0.5; (**b**) the optimized LFM-PC waveforms.

The ambiguity functions of the approximate LFM waveforms designed by SIA in [28] and the final optimized LFM-PC waveforms are demonstrated in Figure 19a and b, respectively. The ambiguity function of our proposed waveforms has a favored thumbtack shape, while that of the SIA-designed waveforms has a serous delay-doppler coupling. In our proposed algorithm, the sidelobes within the doppler frequency range $F_{d}$ have been greatly suppressed. Further research will be continued to design the constrained beampattern with optimized sidelobes at a lower computation load for a wider doppler frequency range.

We further summarize the values of PSL and PAT for the two-main-lobe desired pattern quantitatively in Table 7. The conclusions reflected by these values are similar with those in Table 4. We find that: (1) The real values of the PSL drop from 0.2614 before optimization to 0.1897 after the two stages of optimization, and the corresponding dB values of the PSL drop from −11.6539 dB before optimization to −14.4387 dB after the two stages of optimization. (2) The beampattern matching errors drop from 2.9812 before optimization to 1.6053 after the two stages of optimization. (3) From Stage I to Stage II, the PAT is further reduced at the cost of about 2 dB increase on PSL, which is realized through the mandatory optimization. (4) By comparing the values in Table 4 and Table 7, after two stages of optimization, both the PSL and the PAT for the two-main-lobe scenario is higher than those for the one-main-lobe scenario. This means that it is more difficult to match the two-main-lobe beampattern than the one-main-lobe beampattern. (5) The PSL performance of our designed LFM-PC waveforms is much better than that of the SIA-designed waveforms. The PAT error value of the SIA-designed waveforms seems slightly better than that of our proposed waveforms, which is because the PAT is only a rough metric to describe the matching degree, and cannot judge the goodness of the beampattern completely. From Figure 17, the two mainlobes of the SIA-designed waveforms are not as obvious as those of our proposed waveforms.

## 7. Conclusions

In this paper, the constrained transmit beampattern design problem using the correlated LFM-PC waveforms was considered. The ambiguity function of the LFM-PC waveforms was analyzed, and the superiority of the multicarrier LFM-PC waveform set was verified. Then, based on the temporal and spatial characteristics of the LFM-PC waveform set, a bi-objective optimization problem was formulated to design the transmit beampattern constrained by a good ambiguity function property. The frequency steps, carrier bandwidths, and phase-coded sequences of the LFM-PC waveforms were chosen as the optimization variables. We proposed a two-stage optimization strategy to solve this. In the first stage, the transmit beampattern and sidelobe level were jointly optimized. In the second stage, the transmit beampattern was mandatorily optimized at the slight loss of sidelobe performance. Simulation results demonstrate that by using the LFM-PC waveforms, the desired beampattern can be approached well; the range sidelobes are effectively suppressed; and a thumbtack-shape ambiguity function is obtained.

## Figures and Tables

**Table 1:** Parameter setup for the simulation.

Symbol	Quantity	Value
*M*	transmit antenna number	7
*T*	pulse width	400 μs
*B*	total bandwidth	500 kHz
θ	spatial angle of target	60${}^{\circ }$
Δf	frequency step	40 kHz
$B_{s}$	baseband bandwidth	260 kHz
*Q*	code length	63

**Table 2:** Optimized bandwidths and frequency intervals for the one-main-lobe (/kHz).

Items	$W_{1}$	$W_{2}$	$W_{3}$	$W_{4}$	$W_{5}$	$W_{6}$	$W_{7}$
Stage I							
$B_{LFM,m}$	104.224	1.116	234.896	160.923	235.397	148.47	206.866
$B_{m}$	210.877	157.504	282.812	225.172	283.228	216.448	260
$\Delta f_{m}$	0	31.928	66.137	30.962	14.977	43.474	52.519
Stage II							
$B_{LFM,m}$	135.69	443.136	138.041	162.470	234.948	150.389	206.866
$B_{m}$	207.889	157.5	285.429	226.28	282.854	217.769	260
$\Delta f_{m}$	0	32.189	65.817	31.719	14.967	43.237	52.068

**Table 3:** Optimized phase codes for the one-main-lobe ($/\frac{\pi }{2}$).

Stage I	
$W_{1}$	223222200120100021332113301210121320001333203000120021113001103
$W_{2}$	102012032132200102002020012102220200001320111212201320121120100
$W_{3}$	001000010200013000102021211312002301320132002210012200101012000
$W_{4}$	020010013200001121200020121120131110021320032110201213332133322
$W_{5}$	233203021032120100121212302230130132111020211032011120013330013
$W_{6}$	203320001210132220012213033113201000332012012020333211001222032
$W_{7}$	000013211201331001000202131213200101133130133233001032000023331
Stage II	
$W_{1}$	123220000020112202002103002220121322002232103000022001120200100
$W_{2}$	102212032002200212022020002202220200001120011222100320121010000
$W_{3}$	000001000100023000102010001002022301300002102211020001121210110
$W_{4}$	020010013001012022000020111120120210022320202200201113032031022
$W_{5}$	213003020102121201220211302231130202122011122032001110012330010
$W_{6}$	223220001212032200112212033020202002302010002021313211001222232
$W_{7}$	000213211200131001000200031110000201030110032031212000011021212

**Table 4:** Optimization results for one-main-lobe.

Waveforms	PSL	PSL (dB)	PAT
OFD-LFM	0.4640	−6.6696	2.4220
Random LFM-PC	0.2218	−13.8080	1.9919
LFM-PC of Stage I	0.1457	−16.7308	1.1291
LFM-PC of Stage II	0.1576	−16.0489	0.6677
SIA ξ=0.5	0.6187	−4.1701	0.9999

**Table 5:** Optimized bandwidths and frequency intervals for the two-main-lobe (/kHz).

Items	$W_{1}$	$W_{2}$	$W_{3}$	$W_{4}$	$W_{5}$	$W_{6}$	$W_{7}$
Stage I							
$B_{LFM}$	96.719	335.681	4.163	8.661	136.176	253.396	206.866
$B_{m}$	184.826	370.794	157.555	157.737	208.207	298.355	260
$\Delta f_{m}$	0	12.659	12.661	10.101	28.688	175.397	0.491
Stage II							
$B_{LFM}$	99.787	333.01	4.108	6.977	133.815	257.315	206.866
$B_{m}$	186.45	368.378	157.553	157.654	206.67	301.691	260
$\Delta f_{m}$	0	12.503	12.734	10.927	28.696	174.633	0.503

**Table 6:** Optimized phase codes for the two-main-lobe ($/\frac{\pi }{2}$).

Stage I	
$W_{1}$	211202213212101112310220113330122002103333112333213101223132002
$W_{2}$	320333202010003313200132332213303122023321121313022121313220001
$W_{3}$	131321311300132201000332101120313100131210233013320131333321332
$W_{4}$	213100003233022232211030023322333032132110101230131210221201003
$W_{5}$	121033130112312120131333133302230133203311212003130012000221010
$W_{6}$	111120031201320113212330123131102133213031031033203121321310232
$W_{7}$	003320133123132213131231002013323211321320321333301201310021300
Stage II	
$W_{1}$	100101113202101212010020113330122012103230122303113011222132001
$W_{2}$	010333200000000113100131332213003122021321210300022101010222010
$W_{3}$	220111311001000221110201101000013211031010200010120122330321232
$W_{4}$	213211022000122130200001001012310230022110201000020211220110003
$W_{5}$	221000100001002120002302103100201200102301121210222002000212000
$W_{6}$	010120000201311103001000020130102200011101020100202012300310120
$W_{7}$	000300130120122200031000011010000121200320020130210101200022310

**Table 7:** Optimization results for the two-main-lobe.

Waveforms	PSL	PSL (dB)	PAT
OFD-LFM	0.4640	−6.6696	3.1086
Random LFM-PC	0.2614	−11.6539	2.9812
LFM-PC of Stage I	0.1541	−16.2439	2.1303
LFM-PC of Stage II	0.1897	−14.4387	1.6053
SIA ξ=0.5	0.6912	−3.2081	1.1269
